# Gut Microbiota: A New Challenge in Mood Disorder Research

**DOI:** 10.3390/life15040593

**Published:** 2025-04-03

**Authors:** Giuseppe Marano, Sara Rossi, Greta Sfratta, Gianandrea Traversi, Francesco Maria Lisci, Maria Benedetta Anesini, Roberto Pola, Antonio Gasbarrini, Eleonora Gaetani, Marianna Mazza

**Affiliations:** 1 Unit of Psychiatry, Fondazione Policlinico Universitario A. Gemelli IRCCS, 00168 Rome, Italygreta.sfratta@hotmail.it (G.S.); mbenedetta@hotmail.it (M.B.A.); mariannamazza@hotmail.com (M.M.); 2 Department of Neurosciences, Università Cattolica del Sacro Cuore, 00168 Rome, Italy; 3 Unit of Medical Genetics, Department of Laboratory Medicine, Ospedale Isola Tiberina-Gemelli Isola, 00186 Rome, Italy; 4 Section of Internal Medicine and Thromboembolic Diseases, Department of Internal Medicine, Fondazione Poli-Clinico Universitario A. Gemelli IRCCS, Università Cattolica del Sacro Cuore, 00168 Rome, Italy; roberto.pola@policlinicogemelli.it; 5 Department of Medical and Surgical Sciences, Fondazione Policlinico Universitario A. Gemelli IRCCS, Università Cattolica del Sacro Cuore, 00168 Rome, Italy; 6 Department of Translational Medicine and Surgery, Fondazione Policlinico Universitario A. Gemelli IRCCS, Università Cattolica del Sacro Cuore, 00168 Rome, Italy; 7 Unit of Internal Medicine, Cristo Re Hospital, 00167 Rome, Italy

**Keywords:** gut microbiota, mood disorders, mental health, gut–brain axis, personalized medicine, lifestyle

## Abstract

The gut microbiome has emerged as a novel and intriguing focus in mood disorder research. Emerging evidence demonstrates the significant role of the gut microbiome in influencing mental health, suggesting a bidirectional communication between the gut and the brain. This review examines the latest findings on the gut–microbiota–brain axis and elucidates how alterations in gut microbiota composition can influence this axis, leading to changes in brain function and behavior. Although dietary interventions, prebiotics, probiotics, and fecal microbiota transplantation have yielded encouraging results, significant advances are needed to establish next-generation approaches that precisely target the neurobiological mechanisms of mood disorders. Future research must focus on developing personalized treatments, facilitated by innovative therapies and technological progress, which account for individual variables such as age, sex, drug history, and lifestyle. Highlighting the potential therapeutic implications of targeting the gut microbiota, this review emphasizes the importance of integrating microbiota research into psychiatric studies to develop more effective and personalized treatment strategies for mood disorders.

## 1. Introduction

The gut microbiome is a complex ecosystem comprising trillions of microorganisms, including bacteria, viruses, fungi, and archaea [1]. This intricate microbial community plays crucial roles in digestion, nutrient synthesis, and immune system modulation [2]. Imbalances in microbiota composition, known as dysbiosis, can have systemic effects, contributing to inflammatory, metabolic, and psychiatric disorders. Microbiome modulation is emerging as a promising avenue for addressing complex conditions, including neuropsychiatric disorders, although further clinical evidence is needed to establish its efficacy [3,4].

The gut–brain axis, a bidirectional communication system involving the autonomic nervous system, endocrine system, and immune system, mediates the relationship between the gut microbiota and the central nervous system (CNS) [5]. This interaction enables the microbiota to influence brain function and behavior. Microbial metabolites, such as short-chain fatty acids (SCFAs), bacterial neurotransmitters (e.g., serotonin, GABA), hormones (e.g., cortisol), and immune modulators (e.g., quinolinic acid), facilitate communication [6]. A critical aspect of this interaction is the modulation of biological barriers like the intestinal and blood–brain barriers. Dysbiosis can compromise these barriers, leading to systemic and neuroinflammation, which are closely linked to mood and neuropsychiatric disorders [7].

The gut microbiome plays a significant role in mood regulation and the pathophysiology of psychiatric disorders, including bipolar disorder (BD) and major depressive disorder (MDD). MDD, a leading cause of disability worldwide, has been strongly linked to alterations in gut microbiome composition [8]. While traditionally attributed to imbalances in neurotransmitters like serotonin, dopamine, and norepinephrine, MDD is increasingly recognized as a complex disorder involving dysfunctions of the gut–brain axis [9]. BD, a severe psychiatric condition characterized by alternating episodes of mania and depression, has been increasingly linked to gut microbiota alterations [10,11]. Globally, BD affects approximately 2.4% of the population, with 6–7% of patients at risk of suicide [12]. Despite advances in pharmacological and psychological treatments, many patients remain treatment-resistant, prompting research into novel strategies like microbiome modulation. Studies suggest that intestinal dysbiosis in bipolar patients may disrupt gut–brain axis regulation, contributing to metabolic and immune dysfunctions [13]. Specifically, reduced bacterial diversity and alterations in specific microbial taxa have been linked to the severity of bipolar symptoms, including cognitive deficits and emotional dysregulation [12]. These findings pave the way for innovative interventions aimed at restoring microbial balance to improve bipolar disorder management.

In this review, we explore the potential therapeutic implications of targeting the gut microbiome for the treatment of mood disorders. The paper discusses the potential benefits and challenges of using probiotics, prebiotics, and other microbiome-based therapies to treat mood disorders, focusing on the evidence for their efficacy and potential side effects and safety concerns.

## 2. Gut Microbiota and Mental Health

The intricate relationship between gut microbiota and mental health has garnered significant attention in recent years, highlighting the profound impact of gut microbiome composition on mental well-being [14]. Dysbiosis, or an imbalance in gut microbiota, has been linked to various mental health conditions, including mood disorders [15]. Diets rich in fiber, probiotics, and prebiotics promote a healthy gut microbiome, while high-fat and high-sugar diets can lead to dysbiosis [16]. Over the lifetime, changes in diet, stress levels, and medication use can alter gut microbiota, potentially influencing mental health outcomes. Recent research has explored the potential of dietary interventions and probiotics as therapeutic strategies for mental health disorders, emphasizing the importance of maintaining a balanced gut microbiota for overall mental well-being [17].

### 2.1. Gut Microbiota Composition

The gut microbiome refers to the intricate and diverse community of microorganisms inhabiting the human gastrointestinal tract. These microorganisms, comprising bacteria, archaea, viruses, and eukaryotes, are present in greater numbers and diversity within the gut than in any other part of the body [18]. The ratio of microorganisms to human cells is approximately 1:1 (3.8 × 10^13^ microorganisms to 3 × 10^13^ human cells). However, the genetic content of microbes far exceeds that of human cells, being 100 to 200 times greater [19].

**Figure 1:**
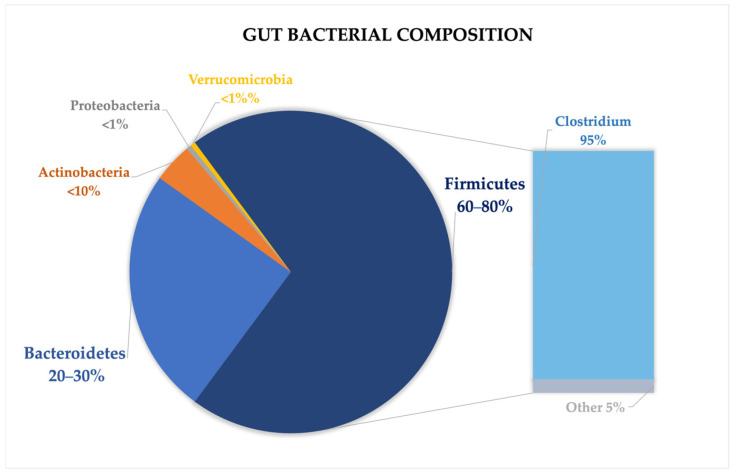
Gut microbiome composition in healthy subjects, highlighting the most prevalent phyla, with a particular focus on Firmicutes.

The gut microbiome is composed of over 1500 species, with *Firmicutes* and *Bacteroidetes* accounting for over 90% of the total microbial population. Other less abundant phyla include *Proteobacteria*, *Actinobacteria*, *Verrucomicrobia*, and *Fusobacteria* [18,20]. The *Firmicutes* phylum encompasses over 200 genera, including well-known ones like *Lactobacillus*, *Bacillus*, *Clostridium*, *Enterococcus*, and *Ruminococcus*. Within this group, the *Clostridium* genus constitutes the vast majority, accounting for approximately 95% of the phylum. Meanwhile, the *Bacteroidetes* phylum is primarily made up of dominant genera such as *Bacteroides* and *Prevotella* [20,21,22] (Figure 1).

The gut microbial community is highly dynamic, having evolved to thrive within the gastrointestinal tract, adapting to conditions such as moisture, temperature, pH, and nutrient availability. Some bacterial genera are considered beneficial symbionts, as they maintain a mutually advantageous relationship with their human host. On the contrary, other genera are identified as potential pathogens, and an imbalance between these groups can heighten the host’s susceptibility to developing diseases. While this balance may differ depending on individual contexts, bifidobacteria and lactobacilli are typically recognized as beneficial bacteria, often included in probiotic supplements [23].

Recent advancements in next-generation sequencing technologies, such as 16S rRNA sequencing, have significantly enhanced our ability to identify and quantify microbial species, opening the doors to personalized therapies in the future [24].

The gut microbiome plays a crucial role in human health. It aids in food digestion, synthesizes essential vitamins, enhances the absorption of minerals, and contributes to the development and modulation of the immune system. Moreover, the gut microbiota play a key role in maintaining mental health balance via the gut–brain axis. This bi-directional communication highlights the significant influence the gut microbiome has on brain function and emotional well-being, potentially contributing to the development of psychiatric disorders [25].

The composition of the gut microbiome varies widely among individuals and can be influenced by numerous factors, including diet, age, genetics, lifestyle, geography, antibiotics, and method of delivery [26].

### 2.2. Microbiota Inhabiting Different Segments of Gastrointestinal Tract

The gastrointestinal (GI) tract is anatomically subdivided in segments including the oral cavity, pharynx, esophagus, stomach, small intestine (further divided into duodenum, jejunum, and ileum), large intestine (cecum, colon, rectum) and anal canal [27]. Each of these regions harbors a distinct microbial environment, influenced by factors such as pH, oxygen levels, host secretions and nutrient availability [20]. The stomach, in particular, presents a hostile environment for most microorganisms due to its strong acidity, which eliminates most external bacteria, resulting in a low bacterial colony count (approximately 10^3^ CFU per g/mL) [26]. Despite this, *Firmicutes* and *Proteobacteria* are the dominant phyla, with *Streptococcus* and *Prevotella* as major genera [28]. Infection with *Helicobacter pylori*, a Gram-negative bacterium, significantly alters the gastric microbiota and can lead to gastritis, ulcers, and gastric cancer [29]. In the duodenum, the rapid transit of food and limited oxygen availability reduce bacterial density and diversity, with a predominance of *Firmicutes* and *Actinobacteria* [26].

In the jejunum, the microbial population becomes more diverse and denser, supporting a variety of Gram-positive aerobes and facultative anaerobes, including lactobacilli, enterococci and streptococci. The large intestine, with its slower transit time and anaerobic conditions, harbors the largest and most diverse microbial community. The microbiome in this region is primarily anaerobic, with *Firmicutes* and *Bacteroidetes* being the most common phyla [26]. Furthermore, longer gut transit times are associated with higher fecal pH, reduced water content, and changes in microbial metabolism. As readily available carbohydrates become scarce in the colon, gut bacteria shift towards fermenting proteins, which can produce harmful byproducts like branched-chain fatty acids (BCFAs) and hydrogen sulfide. These shifts contrast with the beneficial short-chain fatty acids (SCFAs) produced when carbohydrates are more abundant [30].

### 2.3. Lifestyle and Diet

One of the most crucial factors shaping gut microbiota and the immune system is diet [20]. Each dietary component directly affects host health. This influence occurs through interactions with the intestinal epithelial barrier, commensal bacteria, and immune cell phenotypes, shaping pro-inflammatory or anti-inflammatory responses [25]. Among macronutrients, carbohydrates have been most extensively investigated, with dietary fibers receiving particular attention [20,23].

Dietary fibers like galactooligosaccharide and polydextrose act as prebiotics, promoting the growth of beneficial gut bacteria such as *Lactobacilli*, *Bifidobacteria* and *Parabacteroides* [25,31]. These fibers are transformed into SCFAs, which contribute to strengthening the intestinal barrier, mediate systemic anti-inflammatory properties, and play a key role in immune regulation by modulating cytokine production and T-cell activity. SCFAs also influence brain health via the gut–brain axis, interacting with microglia and stimulating enteroendocrine cells to release appetite-regulating peptides like GLP-1 and PYY. Additionally, gut bacteria produce neurotransmitters, such as norepinephrine and dopamine, impacting mood and behavior by engaging with the nervous system [32]. The Mediterranean Diet (MD), characterized to be rich in fiber, healthy fats, and bioactive compounds, supports microbial balance by promoting beneficial bacteria like *Bacteroidetes* and reducing harmful phyla such as *Proteobacteria*. This diet reduces chronic inflammation, enhances immune function, and protects against non-communicable diseases (NCDs). Conversely, the Western Diet (WD), characterized by unhealthy fats, refined sugars, and ultra-processed foods, disrupts gut microbiota, leading to dysbiosis, systemic inflammation, and increased risks for conditions like obesity and type 2 diabetes. These dietary influences underscore the crucial role of gut microbiota in health and disease [19].

Engaging in daily exercise has been shown to enhance gut microbial diversity, increasing the abundance of Firmicutes-associated taxa such as *Clostridiales*, *Roseburia*, *Lachnospiraceae*, and *Erysipelotrichaceae*. This enrichment promotes the production of SCFAs, which strengthen the intestinal barrier by increasing the expression of tight junction proteins in colon epithelial cells. As a result, exercise helps lower mucosal permeability and reduces the production of inflammatory cytokines, contributing to improved gut and mental health [20]. Cataldi et al. found evidence, in both preclinical and human models, that aerobic activities at a moderate and brief intensity can impact the gut microbiota, particularly by reducing inflammation and favoring bacterial community richness. Physical activity seems to promote SCFAs-producing bacteria, favoring the colonization of *Bacteroidetes* phylum and reducing the abundance of *Firmicutes* bacteria, which can positively impact brain function and mental health [33].

### 2.4. Delivery and Milk Feeding

Traditionally, the intestine was considered sterile at birth; however, recent evidence suggests that gut microbiota colonization may begin prenatally through maternal–fetal microbiota transfer during pregnancy. While this hypothesis remains controversial due to the potential for sample contamination and a lack of robust supporting data, the composition and development of the infant gut microbiota are influenced by various prenatal factors, including maternal diet, obesity, smoking status, and antibiotic use during pregnancy [20]. Nevertheless, the mode of delivery and breastfeeding remain the major determinants of early gut colonization [34].

Newborns delivered vaginally acquire a microbiota community that strongly reflects that of their mother’s vaginal microbiota [20,35], including *Parabacteroides* spp., *Bacteroides* spp. (particularly *Bacteroides fragilis* [34]), *Bifidobacterium* spp., and *Escherichia coli* [34]. Additionally, *Lactobacillus*, *Prevotella*, *Sneathia*, and other facultative anaerobes such as *Staphylococcus* and *Streptococcus* also colonize the infant gut [20,35].

Infants delivered via cesarean section primarily acquire bacteria from the hospital environment and the mother’s skin, including species such as *Staphylococcus*, *Corynebacterium*, and *Propionibacterium*, *Escherichia*-*Shigella*, and *Bacteroides* species, which are notably underrepresented in these infants compared to vaginally born infants [20]. Among infant-feeding methods, breastfeeding is often preferred. Breast milk provides essential nutrients, as well as pro-microbial and antimicrobial factors, and promotes the growth of beneficial bacteria, particularly *Bifidobacterium* species [20,36,37]. Maternal microbial transmission occurs both indirectly via milk components like human milk oligosaccharides (HMOs) and secretory IgA and directly through exposure to the milk microbiota. HMOs, the third-largest solid component of breast milk, selectively promote the growth of *Bifidobacterium breve* and *Bifidobacterium bifidum* [34].

Breastfed infants exhibit higher richness and diversity of *Bifidobacterium* spp. compared to formula-fed infants, who harbor less than half the number of *Bifidobacterium* cells. These *Bifidobacterium*-rich microbiota facilitate the fermentation of galactooligosaccharides (GOS) in breast milk, producing SCFAs like acetate and lactate. These metabolites contribute to a decrease in gut pH, thereby enhancing gut health, reflecting the prebiotic effects of HMOs on stimulating *Bifidobacterium* growth. In contrast, formula feeding is associated with a different microbiota profile, characterized by increased colonization by *Escherichia coli*, *Bacteroides* species, and *Clostridioides difficile*. Additionally, β-Palmitate, derived from palmitic acid—the most abundant saturated fatty acid in human milk—has demonstrated prebiotic effects by promoting the growth of *Bifidobacterium* and *Lactobacillus* species [20,38].

While there is substantial evidence linking infant gut dysbiosis to an increased risk of necrotizing enterocolitis and adult-onset NCDs, including obesity, diabetes, cancer, allergies, and asthma [36,39], there is limited research on its association with the development of childhood and adult psychiatric disorders. Future studies should prioritize exploring the potential of prebiotics and probiotics in early life to modulate the gut microbiota and potentially mitigate these risks.

### 2.5. Lifetime Changes

The gut microbiome undergoes significant changes throughout life, influenced by genetic and environmental factors such as race, ethnicity, diet, lifestyle, and drug use [40]. By one year of age, the microbiome is primarily colonized by *Akkermansia muciniphila*, *Bacteroides*, *Veillonella*, *Clostridium coccoides* and *Clostridium botulinum* [20]. By the age of 3–5 years, the microbiota stabilizes, achieving a composition that is 40–60% similar to that of adults [26].

During adulthood, the microbiome remains stable unless disrupted by factors such as dietary changes, antibiotics, stress, or diseases. Aging impacts the gut microbiota through processes like immunosenescence, which reduces immune system functioning, and inflammaging, which causes chronic low-grade inflammation. These changes lead to a reduction in the diversity of beneficial bacteria, such as *Lactobacilli* [19,40], a decline in butyrate-producing species, and an increased presence of potential pathogens, especially in centenarians.

These changes are associated with reductions in the production of SCFA such as acetate, propionate, and butyrate, which play vital roles in maintaining gut health by lowering pH, inhibiting pathogen overgrowth (e.g., *Escherichia coli*), and promoting the growth of beneficial *Firmicutes*. Alterations in SCFA production have been linked to increased frailty in aging populations [40].

## 3. The Role of the Microbiota in Regulating the Gut–Brain Axis

The gut microbiome consists of approximately 100 trillion microbial cells, with *Bacteroidetes* and *Firmicutes* as the dominant groups, representing 75–80% of the total population. Other phyla, including *Proteobacteria*, *Acinetobacteria*, *Fusobacteria*, and *Verrucomicrobia*, are present in smaller proportions but significantly interact with other gut microorganisms, contributing to human health by interfacing with the immune and nervous systems. In healthy individuals, the microbiome maintains a delicate balance with host cell activities, ensuring the physiological homeostasis of the gut–brain axis [41,42,43,44].

The microbiome actively participates in digestion by enhancing nutrient absorption through the intestinal epithelium. It preserves the integrity of the intestinal barrier by regulating cellular metabolism and promoting immune response development [45]. However, disruptions in the regulation of intestinal barrier permeability can trigger innate immune responses, leading to systemic and brain inflammation. This inflammatory state can increase vulnerability to stress and psychiatric disorders. The commensal microbiome plays a crucial role in mitigating epithelial inflammation by coordinating anti-inflammatory responses, producing antimicrobial proteins, defending the epithelial surface with mucus secretion, and repairing intestinal tissue damage [46].

Several factors can influence the composition and functionality of the microbiome, including age, geographic location, diet [47], medication usage, toxins, infectious agents, and host genetics [48]. Additionally, the neonatal microbiome is shaped by the maternal microbiota during childbirth and breastfeeding [49,50].

Recent research suggests that the gut microbiome produces various neurotransmitters. For instance, *Bifidobacterium* and *Lactobacillus* synthesize GABA, enhancing inhibitory brain network pathways. Meanwhile, *Lactobacillus* and *Oscillibacter* increase the expression of tryptophan synthase, boosting serotonin production. The production of neurotransmitters depends on factors such as vagus nerve stimulation and immune system activity. The activation of afferent vagal pathways through G-protein-coupled receptors or histone deacetylases modulates immune cell function, including monocytes, macrophages, neutrophils, dendritic cells, and T-cell recruitment and differentiation [51,52].

The vagus nerve plays a central role in regulating the gut–brain axis due to its extensive network of nerve endings in the intestinal mucosa and submucosa, accounting for approximately 90% of its total nerve endings. It influences gastrointestinal functions such as gastric emptying inhibition and secretion of digestive enzymes, supporting digestion and nutrient absorption [53]. Beyond the gastrointestinal tract, the vagus nerve is integral to memory, emotion, and cognition, as its connections extend to the cerebral cortex, amygdala, and hippocampus [54]. The vagus nerve can detect signals from microbial metabolites via diverse pathways, including afferent sensory mechanisms and receptor networks on its surface, such as serotonin (5-HT) and dopamine receptors, Toll-like receptors (TLR4), and free fatty acid receptors [55]. Disruptions in this signaling system, as evidenced by neuroimaging studies, may lead to functional changes that contribute to the onset of mental health conditions such as substance dependence [56], mood disorders, and eating disorders [56,57].

From the earliest phases of life, the gut microbiota plays an essential function in shaping immune responses at multiple levels. It supports innate immunity by activating gut-associated lymphoid tissue, while interactions between bacterial components and specific receptors—such as TLR9 and inflammasomes—on the surfaces of epithelial and immune cells trigger both localized and systemic immune responses [58]. When the immune system fails to differentiate between self and non-self antigens, it initiates a pathological process that targets the body’s own tissues. This phenomenon, termed autoimmunity or the breakdown of self-tolerance, arises through an adaptive immune response directed against self-antigens. It involves both the innate and adaptive arms of the immune system, disrupting intercellular signaling pathways and contributing to the development of autoimmune diseases (AIDs) [45,59].

At the core of pathogenic recognition are T and B lymphocytes, which orchestrate the process by presenting specific molecular signals on their surfaces, including Pathogen-Associated Molecular Patterns (PAMPs) [45]. During cell death events such as necrosis or apoptosis, endogenous molecules termed Damage-Associated Molecular Patterns (DAMPs) are released. These molecules are detected by pattern recognition receptors (PRRs), initiating a pathological inflammatory cascade [45,59]. Similar mechanisms occur in the gut, where intestinal epithelial cells stimulate immune responses through complexes of Toll-like receptors, Nod-like receptors, and helicases. An autoimmune response is triggered when the immune system becomes aberrantly activated in response to ordinarily harmless stimuli [60,61].

Clinical observations and scientific research increasingly point to a significant association between autoimmune processes and psychiatric conditions [62]. This connection may involve the altered antigenic presentation of brain proteins, molecular mimicry mechanisms, and the generation of autoantibodies that cross-react with neuronal structures. Such processes are implicated in neurological disorders like Parkinson’s disease and multiple sclerosis [63,64].

The onset of autoimmunity is influenced by a combination of factors. While genetic predisposition remains a critical determinant of individual susceptibility, environmental influences—such as stress, exposure to xenobiotics, dysregulation of the microbiota, and infections by pathogens—play a pivotal role in modulating, amplifying, or suppressing autoimmune responses [48,64].

When immune function is compromised, the metabolism of specific metabolites and neurotransmitter precursors—many of which are microbiota-derived—is disrupted. For instance, the metabolism of tryptophan, a precursor of serotonin, is altered under conditions of autoimmune dysfunction. This leads to the increased production of kynurenine, a molecule also synthesized by gut microbes. Accelerated kynurenine metabolism generates kynurenic acid and quinolinic acid, which in turn affect the secretion of GABA and dopamine, as well as synaptic plasticity and function [65,66]. These two metabolites exert distinct effects based on their concentrations. While kynurenic acid at physiological levels offers neuroprotection by antagonizing N-methyl-D-aspartate (NMDA) receptors, elevated levels of quinolinic acid are associated with cognitive impairment due to synaptic dysfunction [67]. Some studies suggest that quinolinic acid’s excitotoxicity may further impair synaptic plasticity [68]. Several kynurenine metabolites have emerged as promising biomarker candidates for mood disorders [69]. The kynurenine/tryptophan (KYN/TRP) ratio is commonly used as a proxy for indoleamine 2,3-dioxygenase (IDO) activity, and elevated levels have been associated with both peripheral inflammation and depressed symptoms [70,71]. Some pharmacological strategies are currently being investigated to target this pathway. The MINDEP trial tested minocycline, an antibiotic with anti-inflammatory and neuroprotective properties, as an augmentation strategy in treatment-resistant depression (TRD). Although minocycline did not significantly alter kynurenine metabolites, it was associated with a notable reduction in suicidal ideation [71].

## 4. Modulating Mood Through the Gut: The Role of the Microbiota

Gut bacteria interact with the central nervous system (CNS) through the gut–brain axis, a multidirectional communication network involving the immune system, endocrine signaling (e.g., the hypothalamic–pituitary–adrenal [HPA] axis), the vagus nerve, and microbial production of neuroactive compounds [43,72,73].

Mood disorders are characterized by complex interactions between genetic predispositions, environmental factors, and disruptions in neurochemical pathways. Increasing evidence supports the hypothesis that the gut microbiome acts as a mediator in this intricate interplay [74,75,76]. Alterations in microbial composition and diversity have been observed in individuals with mood disorders, correlating with clinical symptoms such as inflammation, neurotransmitter dysregulation, and neurocognitive deficits [77]. For instance, BD patients exhibit a distinct microbial profile marked by reduced *Faecalibacterium* abundance and an increased representation of pro-inflammatory species such as *Actinobacteria* and *Enterobacteriaceae*. Similarly, MDD is associated with decreased microbial diversity and shifts in taxa linked to inflammation and impaired neurotransmitter synthesis [78,79,80].

The microbiome’s ability to synthesize and regulate neurotransmitters—including serotonin, dopamine, and GABA—underscores its relevance to mood disorders. Additionally, gut microbes influence systemic inflammation and CNS function through pathways such as Toll-like receptor (TLR) signaling and the modulation of tryptophan metabolism via the kynurenine pathway. The dysregulation in these pathways has been implicated in the onset and progression of mood disorders, highlighting the therapeutic potential of targeting the gut microbiota to alleviate psychiatric symptoms [81,82,83,84].

The gut microbiota composition in individuals with BD appears to differ significantly from that of healthy subjects (Table 1). Specifically, BD patients exhibit a reduced abundance of *Faecalibacterium*, with greater reductions correlating with more severe symptoms, including pronounced sleep disturbances and psychotic episodes [75].

Additionally, BD patients show increased representation of the phylum *Actinobacteria*, particularly *Coriobacteria* [85], along with elevated levels of Gram-negative bacteria such as *Prevotella* and *Enterobacter species*, and Gram-positive bacteria including *Atopobium Cluster*, *Clostridium*, and *Flavinofractor* [74,81]. Furthermore, gut microbiome composition varies not only between BD patients and healthy individuals, but also between BD type 1 and type 2 subgroups. For example, *Prevotella* is more prevalent in type 1 BD, whereas *Collinsella* is more abundant in type 2 BD [74].

Emerging evidence underscores the role of the gut microbiota in influencing neurotransmitter production in BD through the synthesis of neuroactive compounds. One such compound, kynurenine, inhibits 5-HT synthesis and produces metabolites like hydroxykynurenine, which are neurotoxic and interfere with neurotransmitter function [65]. The immune system may exacerbate disruptions in the kynurenine pathway, altering the secretion of neurotransmitters such as dopamine and GABA [66]. These alterations are significant given the strong link between BD onset and GABAergic system dysregulation [86]. Certain commensal microbes within the microbiota also contribute directly to neurotransmitter production. For instance, *Lactobacillus* and *Bifidobacterium* can synthesize GABA, while norepinephrine is produced by *Bacillus*, *Escherichia coli*, and *Saccharomyces*. Similarly, serotonin is generated by *Candida*, *Streptococcus*, *Enterococcus*, and *Escherichia*, dopamine by *Bacillus* and *Serratia*, and acetylcholine by *Lactobacillus* [81].

The gut microbiome interacts with innate immunity through TLRs, a family of pattern recognition receptors expressed on immune cells, neurons, and glial cells. TLRs recognize a broad range of microbial antigens, including peptidoglycans, lipoteichoic acid, and lipoproteins from Gram-positive bacteria, as well as lipopolysaccharides (LPS) from Gram-negative bacteria. Additionally, TLRs detect bacterial DNA, flagellin, and viral or fungal components, contributing to immune surveillance. The activation of these receptors triggers intracellular signaling cascades that lead to the release of pro-inflammatory cytokines, such as IL-6, IL-1α, IL-1β, and TNF-α, promoting neuroinflammation and brain dysfunction [87,88]. Another mechanism linking the gut microbiota to BD pathogenesis involves synaptic pruning, a process essential for neuronal connectivity. The microbiota-mediated modulation of microglial cells can impair synaptic pruning, a process critical for neuronal connectivity. Neuroimaging studies have demonstrated that deficits in synaptic pruning, particularly in the ventral prefrontal cortex and limbic regions, may result from gut microbiota imbalances in BD patients [89]. Similar to psychotic disorders, irritable bowel syndrome (IBS) and other gastrointestinal conditions are frequently reported in individuals with mood disorders, complicating their clinical management. These patients often experience heightened rates of anxiety and depression [81]. There is also evidence of an indirect relationship between mood disorders and intestinal autoimmune diseases, such as celiac disease. Notably, psychological support that improves mental health outcomes has been associated with the better management of celiac disease [90].

BD patients commonly exhibit intestinal inflammation during mood episodes, characterized by elevated levels of pro-inflammatory cytokines [91,92,93]. Alterations in tryptophan metabolism, particularly along the kynurenine pathway, also appear to contribute to BD pathogenesis [94]. Tryptophan metabolism yields excitatory neuroactive compounds such as kynurenic acid, which antagonize NMDA receptors. Excessive concentrations of these metabolites have been identified in BD patients [95].

Alcohol use further complicates the intersection between mood and gastrointestinal disorders. Alcohol consumption, which increased during the COVID-19 pandemic due to social isolation and stress, can disrupt the gut microbiota. Conversely, a healthy microbiome may mitigate liver diseases, such as steatosis, that are linked to alcohol use [96,97]. Antibiotic use also represents an intriguing connection between gut microbiota and BD. Although rare, antibiotics have been reported to induce manic episodes in BD patients, a phenomenon sometimes referred to as “antibiomania” [98]. Interventions targeting the gut microbiota through dietary modifications have shown promise in BD management. Diets rich in antioxidants, plant-based fibers, and B vitamins may reduce depressive symptoms [99], while short-chain fatty acids have been linked to enhanced cognitive function, neurogenesis, and synaptic plasticity [100].

Several studies have turned attention toward the brain–gut–immune system axis. Evidence indicates that gut microbiota can influence stress responses and external stimuli through the modulation of the hypothalamic–pituitary–adrenal (HPA) axis. The dysregulation of this axis is linked to elevated cortisol levels and an increase in pro-inflammatory molecules, both of which are associated with depression and anxiety [72,73,101]. This pro-inflammatory state may, in turn, disrupt gastrointestinal homeostasis, leading to systemic inflammation. For instance, heightened circulating cortisol and inflammatory cytokines can increase intestinal permeability, enabling Gram-negative bacteria to enter the bloodstream and contribute to chronic central nervous system inflammation. This cascade has implications for emotional regulation and mood disorders [72,102]. Such findings reinforce the strong link between microbiota-driven inflammation and psychiatric conditions like anxiety and depression, as observed with irritable bowel syndrome (IBS) [103].

Research indicates that the gut microbiome composition in MDD patients differs significantly from that of healthy controls (Table 2).

Depressed individuals typically exhibit reduced microbial alpha and beta diversity. Specifically, reductions in *Firmicutes*, *Bacteroides*, and *Proteobacteria* are observed alongside increased levels of *Actinobacteria*, *Fusobacteria*, *Prevotellaceae*, and *Lachnospiraceae* [104]. Other changes include diminished concentrations of *Bifidobacterium*, *Lactobacillus*, *Faecalibacterium*, and *Ruminococcus*, as well as higher levels of *Proteobacteria*, *Bacteroides*, and *Prevotella* [104]. Some studies have reported an increase in *Flavonifractor* and a depletion of *Coprococcus* and *Dialister* in depressive patients, while other studies identify associations between *Faecalibacterium*, *Alistipes*, *Ruminococcus*, and MDD [105]. Across recent reviews, a consistent finding is the overrepresentation of pro-inflammatory bacterial species, such as *Actinobacteria* and *Enterobacteriaceae*, and a decrease in protective taxa like *Faecalibacterium* and *Firmicutes* [79,80]. This microbial profile is further influenced by environmental factors, including diet [106], geographic location [107], genetic predisposition [108], and age [109].

Dopamine, a key neurotransmitter involved in reward pathways, is synthesized by gut bacteria and serves as a precursor for catecholamines like epinephrine and norepinephrine, which regulate arousal, cognition, and memory [84]. The dysregulation of these neurotransmitters has been observed in both unipolar and bipolar depression, with elevated norepinephrine levels detected in the plasma and urine of affected individuals [110]. Dopamine plays a critical role in depression pathogenesis. Studies demonstrate that dopamine antagonists worsen depressive symptoms, while dopamine agonists produce antidepressant effects [111]. Dopamine reuptake inhibitors, such as bupropion and venlafaxine, leverage this mechanism to alleviate depressive symptoms [112]. Additionally, elevated levels of homovanillic acid, a dopamine metabolite, have been identified in the mesolimbic and mesostriatal regions of MDD patients treated with transcranial magnetic stimulation (TMS) [113]. The dopaminergic system is closely tied to the microbiome, as demonstrated by its regulation through the HPA axis [114], immune system [115], and vagus nerve [116]. Notably, vagus nerve stimulation increases dopamine levels in the brain [117]. Elevated dopamine and norepinephrine levels promote the growth of pathogenic bacteria, such as *Escherichia coli* O157:H7 (EHEC), enhancing virulence factors like motility and biofilm formation [118]. Similar effects have been observed for *Klebsiella pneumoniae*, *Pseudomonas aeruginosa*, and *Staphylococcus aureus* in vitro. Conversely, the gut microbiome regulates catecholamine synthesis, influencing the growth of bacteria such as *Serratia*, *Morganella*, *Klebsiella*, *Escherichia*, and *Lactobacillus*, which produce dopamine [119].

Recent studies in murine models demonstrate that the antibiotic-induced depletion of gut bacteria increases levels of dopamine precursors, such as levodopa and homovanillic acid (HVA), in the prefrontal cortex. Simultaneously, reductions in HVA are observed in the hippocampus and in the HVA/dopamine ratio in the amygdala and striatum [120]. Other research highlights the beneficial role of commensal bacteria in modulating the dopaminergic system. For instance, the administration of *Lacticaseibacillus paracasei* PS23 reduced levels of dopamine metabolites (e.g., DOPAC and HVA) in the hippocampus of stress-exposed mice without altering dopamine levels [121,122,123].

Similar to dopamine, 5-HT is significantly influenced by gut microbiota. Over 90% of the body’s serotonin is synthesized in the gut, with production attributed to bacteria such as *Candida*, *Escherichia*, *Streptococcus*, *Enterococcus*, *Klebsiella pneumoniae*, *Lactiplantibacillus plantarum*, and *Morganella morganii* [124]. Microbial serotonin production is regulated by butyrate, a short-chain fatty acid that stimulates enterochromaffin cells to produce and release serotonin [124].

## 5. Therapeutic Approaches

The gut microbiota plays a crucial role in maintaining overall health, influencing various physiological processes, including digestion, immune function, and even mental health. Recent advances in therapeutic approaches have focused on harnessing the potential of the gut microbiota to treat a range of conditions. The integration of diet and prebiotics, psychobiotics, fecal microbiota transplantation, and frontiers of innovation represents a comprehensive strategy for addressing gut-related health issues. By combining these approaches, researchers aim to restore and maintain a healthy microbial balance, ultimately improving both physical and mental health outcomes.

### 5.1. Diet and Prebiotics

A growing body of evidence highlights diet as a powerful modulator of gut microbiota composition, with significant implications for mental health, particularly mood disorders such as depression [125]. By promoting beneficial gut bacteria and their associated metabolites, dietary interventions have the potential to enhance mental well-being (Table 3).

WD have been linked to a loss of microbial diversity and gut dysfunction, while healthier dietary patterns, such as the Mediterranean diet, foster gut microbial diversity and beneficial bacteria, which are associated with improved mental health outcomes. Polyphenol- and fiber-rich foods, abundant in Mediterranean diets, enhance SCFA production, reducing inflammation and improving brain function. These beneficial effects of diet on the gut–brain axis suggest that whole-dietary approaches may provide effective strategies for preventing and managing mood disorders through microbiota modulation [126,127]. A recent study by Park et al. has explored the impact of flavonoid-rich orange juice consumption on the gut microbiome and depressive symptoms in 40 young adults aged 20–30. The group consuming flavonoid-rich orange juice (FR) exhibited a significant increase in the abundance of *Lachnospiraceae* and *Bifidobacterium* family compared to the group receiving an equicaloric flavonoid-low orange cordial drink, along with a decrease in the relative abundance of *Clostridium*. Notably, the abundance of *Lachnospiraceae* was positively correlated with serum BDNF levels. These findings suggest that flavonoid-rich orange juice can modulate the gut microbiome by increasing the abundance of *Lachnospiraceae* and *Bifidobacterium*, which may contribute to potential improvements in depression symptoms [128]. Nevertheless, clinical studies in this field are still limited. While animal models offer valuable insights, their findings may not directly translate to humans due to variations in diet formulations and intake levels, and underlying biology. Clinical research is further complicated by individual variability (age, gender, genetics, and baseline microbiota), differences in gut microbiome composition, and methodological limitations in dietary assessment and intervention design. The lack of standardized protocols for dietary assessment further hinders efforts to establish reliable and reproducible connections between diet, microbiota, and mental well-being [23,126].

The current definition of “prebiotic” was established in 2017, when an expert panel from the International Scientific Association for Probiotics and Prebiotics (ISAPP) revised the previous definition to “a substrate that is selectively utilized by host microorganisms, conferring a health benefit”, thereby expanding the potential range of compounds and their effects [132,133]. Prebiotics are primarily composed of carbohydrates, with oligosacchaides (OSCs) being the most common type. Fructans, like inulin and fructo-oligosaccharides, promote the growth of *Bifidobacteria*, although the chain length of fructans plays a role in determining which bacteria are affected [132,134]. Galacto-oligosaccharides (GOS), derived from lactose, promote the growth of *Bifidobacteria* and *Lactobacilli*, with some types, like lactulose-derived GOS, also considered prebiotics. Resistant starch (RS), a type of starch that resists digestion in the upper gut, promotes the production of butyrate and supports certain *Firmicutes* bacteria. Polydextrose, a glucose-derived oligosaccharide, along with pectic oligosaccharides (POS) from pectin and non-carbohydrate compounds like cocoa-derived flavanols, which stimulate lactic acid bacteria, are also regarded as potential prebiotics [132]. Both probiotics and prebiotics can positively impact the gut–brain axis, suggesting their ability to interfere with mental health disorders. A recent systematic review by Ribera et al. [135] included 42 studies (34 RCTs) examining the effects of prebiotics, probiotics, synbiotics, and fermented foods on 2089 participants diagnosed with DSM/ICD mental disorders, such as MD, schizophrenia, and BD. The review encompasses a large number of studies with diverse methodologies and outcome measures, providing a broad perspective on the potential effects of psychobiotics in psychiatric disorders.

While there is some evidence suggesting that these interventions may be helpful, particularly for depression, more research is needed to draw definitive conclusions [135]. Thompson et al. demonstrated that a prebiotic diet enriched with GOS and polydextrose (PDX) consistently and robustly altered the gut microbiome across two research sites. The prebiotic diet increased the abundance of beneficial bacteria like *Bacteroides* and *Parabacteroides*, while reducing potentially harmful genera. Additionally, the diet altered the profile of microbially modified bile acids, specifically decreasing deoxycholic acid. These effects were consistent over time and between the two study sites, demonstrating the potential of prebiotics to promote a health-supporting gut microbiome and improve metabolic function [129].

Tarutani et al. [130] verified the effects of 4G-beta-D-Galactosylsucrose (LS), a prebiotic selectively utilized by *Bifidobacterium* in patients with depressive episodes. While LS consumption did not significantly improve overall depression scores as measured by the Montgomery–Åsberg Depression Rating Scale (MADRS), it tended to improve global self-efficacy (GSES) and quality of life (WHOQOL-BREF). Additionally, 16S rRNA gene sequencing revealed increases in microbial diversity and the relative abundance of *Bifidobacterium* in some participants. These findings suggest that LS may have potential as an adjunctive therapy for depression, particularly in combination with medications and psychotherapy [130]. Conversely, the clinical study by Vaghef-Mehrabani et al. [131] does not align with previous findings. The 8-week prebiotic inulin supplementation in women with obesity and depression shows no significant impact on weight, depression scores, gut permeability, or inflammation biomarkers compared to the placebo group. The lack of statistically significant results could be due to several factors. First of all, the dosage of the prebiotic and the duration of the treatment may not have been adequate. The absence of gut microbiome profiling at the beginning of the study may have contributed to a lack of information necessary for a more targeted intervention. Additionally, the patients have mild to moderate depressive symptoms, and the baseline levels of inflammatory biomarkers are not far outside the normal range, limiting the potential for significant improvements. Although not statistically significant, the results suggest that inulin may enhance satiety and reduce energy intake, potentially supporting long-term weight management and psychological well-being. Further research is required to determine the optimal type, dosage, and duration of prebiotic supplementation for individuals with coexisting depression and obesity.

### 5.2. Psychobiotics

Probiotics, defined by ISAPP in 2013 as “live microorganisms that, when administered in adequate amounts, confer a health benefit on the host” [136], have long been recognized for their role in enhancing gut health and modulating the immune system [137,138,139]. The term probiotic comes from the Latin pro and the Greek *β_lO_σ*, literally meaning “for life”, and the modern concept traces back to Metchnikoff’s early 20th-century observations linking fermented dairy products such as yogurt to improved longevity [140,141]. Recently, the scope of probiotics has expanded with the emergence of psychobiotics—a novel class of probiotics specifically targeting mental health by influencing the gut–brain axis [141]. Psychobiotics impact neurological function through various pathways, including the modulation of microbial composition, the regulation of neurotransmitters, the activation of the immune system and the production of specific microbial metabolites. These microorganisms normalize pro-inflammatory cytokine levels, increase anti-inflammatory cytokines like IL-10, and contribute to immune homeostasis and reduced HPA axis activation [137,142]. The stress-related activation of corticotrophin-releasing hormone (CRF) receptors disrupts gut function, leading to symptoms such as increased gut permeability and altered motility. Psychobiotics mitigate these effects by modulating CRF pathways, as seen in experimental models [142,143]. Additionally, SCFAs derived from psychobiotics, such as acetate, butyrate, and propionate, not only sustain gut health, but also stimulate hormone release and modulate nervous system activity.

Furthermore, psychobiotics promote the synthesis of neurotransmitters through bacterial metabolism. Specific genera, such as *Bifidobacterium*, stimulate GABA production, while *Bacillus* facilitates dopamine and noradrenaline synthesis. Lactobacilli are involved in GABA and acetylcholine production, while *Escherichia* stimulates serotonin and noradrenaline release. This broad spectrum of neurotransmitter regulation underscores the potential use of psychobiotics in addressing mood disorders like anxiety and depression [142]. Together, these mechanisms highlight the potential of psychobiotics as innovative tools for managing mental health disorders, particularly mood disorders, supporting their integration into therapeutic strategies targeting the gut–brain axis. Recent clinical trials and meta-analyses exploring the effects of probiotics on mood disorders, particularly depression and cognition, have yielded varied but promising results (Table 4).

A 2018 meta-analysis by Xiang Ng et al. [144] reviewed 10 randomized controlled trials (RCTs) involving 1349 participants to examine the impact of probiotics on mood and depressive symptoms. While probiotics did not show a significant overall effect on mood (SMD = −0.128, *p* = 0.059), subgroup analysis revealed a notable benefit for individuals with mild to moderate depression (SMD = −0.684, *p* = 0.029). Studies are often heterogeneous in terms of population and study design. In fact, only a few of the included trials specifically recruited patients with clinically diagnosed MDD. Furthermore, depression can manifest in different subtypes, each with distinct clinical symptoms and behavioral patterns. For instance, the atypical depressive subtype may present with increased appetite and weight gain, rather than the typical loss of appetite, potentially resulting in a different gut microbiota composition. This highlights the need for tailored interventions based on individual symptom profiles. Additionally, the use of multi-strain probiotic formulations complicates the identification of the specific strain responsible for clinical benefits. Since not all strains exert the same effects across individuals, the combinations may increase the likelihood of achieving a general therapeutic effect. While *Bifidobacterium breve* shows promising results on depressive symptoms [145], *Lactobacillus* spp. have an effect on cognitive function [100,151]. It is important to consider variable factors such as probiotic strains, dosages, and treatment durations, suggesting further research focusing on clinically diagnosed MDD patients. Probiotics may be useful as an adjunctive therapy, particularly for individuals with mild to moderate depressive symptoms, but should not replace traditional antidepressants [144]. In support of this, several other trials have demonstrated the antidepressant-like potential of specific probiotic strains [145,149]. For example, Tian et al. [145] highlighted how *Bifidobacterium breve* CCFM1025 significantly improve depressive symptoms in MDD patients. In that randomized clinical trial, 45 MDD patients were randomly assigned to receive either maltodextrin or 10^10^ CFU of freeze-dried CCFM1025 daily for four weeks. The CCFM1025 group experienced significant reductions in depressive symptoms, as measured by the HDRS-24 and MADRS, compared to the placebo group. Additionally, the probiotic reduced serum serotonin turnover and influenced gut microbiome composition, with changes in tryptophan metabolism linked to improvements in both emotional and gastrointestinal symptoms. These findings suggest that *Bifidobacterium breve* CCFM1025 may be a promising psychobiotic for treating depression and related gastrointestinal disorders [145].

Casertano et al. [149] investigated the effects of a probiotic formulation containing *Levilactobacillus brevis* P30021 and *Lactiplantibacillus plantarum* P30025 on stress, cognitive performance, and mood. While the probiotic did not significantly affect cognitive performance or overall depressive symptoms as measured by the Depression Anxiety Stress Scale (DASS-42), it notably reduced rumination, a key symptom of depression, as assessed by the Leiden Index of Depression Sensitivity-Revised (LEIDS-r). Additionally, the probiotic group showed significant increases in the abundance of *L*. *plantarum* (*p* = 0.009) and *L. brevis* (*p* = 0.004) compared to the placebo group. These findings suggest that this probiotic formulation may have the potential to alleviate negative mood, particularly rumination, and may be a useful adjunct therapy for individuals with depression [149].

On the other hand, Zhang et al. [148] conducted a study to examine the effects of *Lacticaseibacillus paracasei* strain Shirota (LcS) on constipation in patients with depression. While the LcS group showed greater improvement in specific constipation symptoms, there were no significant differences in overall constipation scores or depressive symptoms compared to the placebo group. However, the LcS intervention led to changes in gut microbiome composition, increasing beneficial bacteria such as *Adlercreutzia*, *Megasphaera*, and *Veillonella*, while reducing bacteria linked to mental illness, along with reduced levels of the inflammatory marker IL-6 (*p* < 0.05). These results suggest that while LcS may not directly impact depressive symptoms, it may influence gut health and inflammation, which are linked to mental health [148]. In terms of brain function, other studies have examined how probiotics affect neural connectivity [146,150,153].

Also, Chahwan et al.’s study [150] demonstrated that probiotics could reduce cognitive reactivity in depressed patients. Improvements in emotional regulation and cognitive function were observed in those with mild to moderate depression who achieved remission in depressive symptoms. The participants in the probiotic group received two sachets each day containing 2 g of *Bifidobacterium bifidum* W23, *Bifidobacterium lactis* W51, *Bifidobacterium lactis* W52, *Lactobacillus acidophilus* W37, *Levilactobacillus brevis* W63, *Lacticaseibacillus casei* W56, *Ligilactobacillus salivarius* W24, *Lactococcus lactis* W19, and *Lactococcus lactis* W58 for eight weeks, showing increased positive effects on depressive symptoms, moving patients from clinical depression to no diagnosis. Notably, probiotics significantly reduced cognitive reactivity, particularly in participants with mild/moderate depression (*p* = 0.01), despite not significantly altering overall gut microbiota composition. However, there was a finding that *Ruminococcus gnavus* bacteria levels were linked to depression scores [150].

Rudzki et al. (2019) [151] found that the probiotic *Lactiplantibacillus plantarum* 299 v, when combined with SSRI treatment, led to cognitive improvements and a reduction in kynurenine concentrations in patients with MDD. This suggests that probiotics could potentially aid in both cognitive function and mood regulation in depression. Also, in Schneider et al.’s [146] study, high-dose multi-strain probiotic supplements showed interesting results on cognitive function during a working memory task. The probiotic formulation contained eight strains: *Streptococcus thermophilus*, *Bifidobacterium breve*, *B. lactis*, *L. acidophilus*, *L. plantarum*, *L. paracasei*, *L. delbrueckii* (*reclassified as L. helveticus*), and *Lactococcus lactis*. The probiotic group showed significant improvements in immediate recall on the Verbal Learning Memory Test (VLMT) compared to the placebo group (*p* = 0.037), suggesting potential improvements in hippocampal function. While no significant differences were observed in other cognitive measures or BDNF levels, these findings suggest that high-dose probiotics could offer a promising therapeutic strategy for cognitive dysfunction in depression, despite the study’s modest sample size and specific formulation [146].

Yamanbaeva et al. [147] further investigated this topic, examining the effects of four-week probiotic supplementation on fronto-limbic brain structure, function, and perfusion in patients with depression using a multimodal neuroimaging approach. The probiotic group showed the stabilization of mean diffusivity (MD) in the right uncinate fasciculus (UF), which correlated with improvements in depressive symptoms, while the placebo group showed increased MD. Neuroimaging analysis also revealed changes in resting-state functional connectivity (rsFC) between the amygdala and the superior parietal lobule, with the probiotic group exhibiting enhanced connectivity, which was linked to cognitive and emotional improvements. Additionally, the probiotic group demonstrated reduced hippocampal activation during working memory tasks, suggesting potential improvements in hippocampal function. While no significant changes in blood perfusion in the amygdala were observed, these findings suggest that four weeks of probiotic supplementation may influence neural mechanisms related to depression, particularly within the fronto-limbic network, and could serve as a promising adjunctive therapy for improving cognitive and emotional symptoms [147].

While the evidence for probiotics as a standalone treatment for depression remains inconclusive, several trials suggest that probiotics can improve depressive symptoms, cognitive function, and gut health, particularly in individuals with mild to moderate depression. These studies support the potential for probiotics as adjunctive therapies in managing depression and associated cognitive impairments, though larger and longer-term studies are needed to confirm these findings. Probiotics, particularly those influencing gut microbiome composition and tryptophan metabolism, show promise in both alleviating depression and improving cognitive outcomes. Research on probiotic supplementation in patients with BD remains limited compared to unipolar depression, but emerging studies suggest potential benefits for cognitive function and gut health, even though their impact on psychiatric symptoms is less clear [100,152,154]. As observed in the pilot study by Reininghaus et al. [100], the results showed significant improvement in cognitive performance, specifically in attention, psychomotor processing speed, and executive functions, in 20 euthymic BD patients over three months. This study highlights the potential role of probiotics in mitigating cognitive dysfunction, a common and debilitating feature of BD. However, the study’s limitations, including the lack of a control group and small sample size, necessitate larger and more rigorous trials. Subsequent research by Reininghaus et al. [154] and Borkent et al. [152] in 2024 further explored this topic by conducting double-blind, placebo-controlled trials, examining the effects of probiotics on BD and schizophrenia spectrum disorder (SSD) patients. While neither study observed notable improvements in overall psychiatric symptoms, both reported a borderline significant improvement in verbal memory in participants receiving probiotics. Furthermore, probiotics demonstrated positive effects on markers of intestinal permeability and inflammation, notably reducing levels of zonulin and alpha-1 antitrypsin. Both studies also noted the safety and tolerability of probiotics, with limited adverse events reported in both treatment and placebo groups. Taken together, these findings suggest that probiotics may serve as a valuable adjunctive therapy for BD, particularly for addressing cognitive domains like verbal memory and improving gut health. Future research should explore the long-term consequences of probiotic supplementation, including its wider mental health effects and the biological mechanisms underlying these changes.

### 5.3. Fecal Microbiota Transplantation

Fecal microbiota transplantation (FMT) is an emerging therapeutic technique that involves transferring stool from a healthy donor into the gastrointestinal tract of a recipient. This procedure aims to restore a balanced and diverse gut microbiome [155,156]. In recent years, FMT has gained significant attention primarily for its efficacy in treating *Clostridioides difficile* infections (CDI), especially in cases refractory to conventional treatments. The cure rate for recurrent or refractory CDI is remarkably high, approaching 90%, surpassing the effectiveness of prolonged antimicrobial therapy [156]. By reintroducing beneficial bacteria, FMT replenishes microbial diversity and counteracts dysbiosis, a state of microbial imbalance in the gut. Beyond CDI, FMT is being explored as a potential therapeutic intervention for a range of other conditions, including irritable bowel syndrome (IBS), inflammatory bowel disease (IBD), and even neurological and psychiatric disorders such as depression and autism spectrum disorder (ASD) [157].

Zhang et al. [158] reviewed the key mechanisms through which FMT alleviates depression in preclinical studies by modulating the gut–brain axis. Studies highlight the role of the Sigma-1 Receptor (Sig-1R), where fecal microbiota from Sig-1R knockout mice induced depressive behaviors in healthy mice, demonstrating the gut microbiome’s influence on depression via the cAMP/CREB/BDNF signaling pathway. Additionally, the NLRP3 inflammasome, a regulator of inflammation, has been linked to depression, and FMT from NLRP3 knockout mice alleviated depression-like behaviors by modulating immune responses and improving astrocyte function. Furthermore, FMT has been shown to reduce systemic inflammation and lower levels of cytokines like IL-1β and IL-18, supporting the idea that altering gut microbiota composition can reduce neuroinflammation and improve depressive symptoms [158]. These findings underscore the potential of FMT as a therapeutic approach for depression, recognizing the bidirectional communication between the gut microbiome and the brain. Recent preclinical studies have provided valuable insights into how FMT may alleviate depression-like symptoms [159,160].

Cai et al. [160] found that FMT in rats subjected to chronic unpredictable mild stress (CUMS) significantly improved depressive behaviors. These effects were linked to the restoration of neurotransmitter balance in the hippocampus, particularly serotonin and GABA, which are essential for regulating mood and anxiety. The microbiota composition in the CUMS+FMT group was more similar to that of the control group than to the CUMS group, suggesting that FMT effectively restored a more balanced gut microbiome. Specifically, FMT increased the abundance of beneficial bacteria such as *Firmicutes* and *Bacteroidetes*, which are known to play a crucial role in energy metabolism, immune regulation, and gut–brain communication. The restoration of a healthy microbiome likely contributed to the improvement in both depressive behaviors and gastrointestinal dysfunction observed in the rats [160]. In line with these findings, Rao et al. [159] examined the effects of FMT in a rat model of stress-induced depression. The researchers reported that FMT alleviated depression-like behaviors by restoring serotonin levels in the brain, particularly in regions like the prefrontal cortex and hippocampus. Furthermore, FMT reduced glial cell activation and inhibited neuroinflammatory pathways, such as the NLRP3 inflammasome, suggesting that FMT’s antidepressant effects may involve both neurochemical and anti-inflammatory mechanisms.

Zheng et al. [161] explored the relationship between gut microbiome dysbiosis and MDD. They found that transplanting fecal microbiota from depressed patients into germ-free mice induced depression-like behaviors in the animals, further supporting the idea that dysbiosis may play a direct role in the pathophysiology of depression. Behavioral tests were conducted to evaluate anxiety-like behavior, memory performance, and depression-like behavior in germ-free (GF) mice, devoid of gut microbiota, and specific pathogen-free (SPF) mice. Metagenomic and metabolomic analyses were performed on mice samples to examine the impacts of the gut microbiome on host metabolism. GF mice exhibited decreased immobility time in the forced swimming test compared to SPF mice, suggesting a potential link between gut microbiota and depression-like behavior. Most human studies on FMT and mood disorders focus on gastrointestinal diseases like IBS, Crohn’s disease, and ulcerative colitis. These studies explore FMT’s potential to restore gut microbiome balance (eubiosis), aiming to improve both intestinal and psychological symptoms, recognizing the strong connection between the gut and the brain [162].

**Figure 2:**
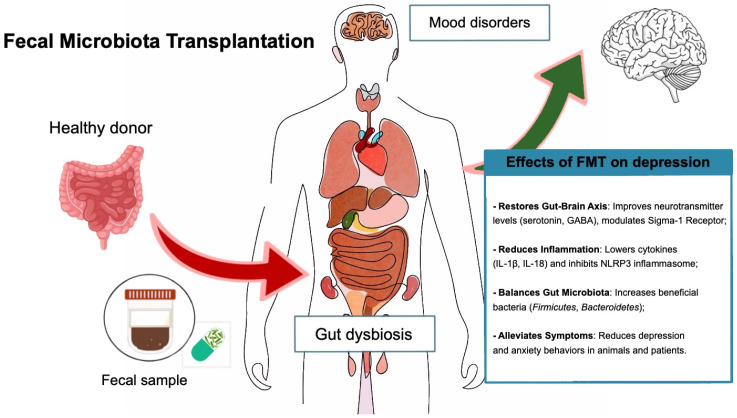
Effects of fecal microbiota transplantation on depression.

For instance, Guo et al. (2021) [163] demonstrated that FMT improved both gastrointestinal and psychiatric symptoms in patients with IBS and comorbid anxiety and depression. Their randomized controlled trial involved 18 patients diagnosed with IBS-D, anxiety, and depression (using HAM-A and HAM-D assessments), who were randomly assigned to either the FMT therapy group or the control group. The FMT group received oral enteric capsules containing fecal microbiota transplantation, while the control group received empty capsules. The FMT treatment was administered in three doses (one every two days, with 30 capsules each time), and symptom assessments were conducted at 1, 8, and 12 weeks after treatment. After receiving oral FMT through enteric capsules, patients experienced significant improvements in IBS severity, anxiety, and depression. These were associated with changes in gut microbiota composition, including an increase in beneficial bacteria like *Bacteroidetes* and *Firmicutes*, which are believed to influence the gut–brain axis and modulate mood [163] (Figure 2).

However, while preclinical findings are promising, clinical evidence remains limited, and more rigorous trials are needed to confirm FMT’s efficacy in treating mood disorders, especially bipolar disorders. Current evidence is predominantly focused on depression, anxiety, and ASD, leaving other psychiatric conditions unexplored. According to Wang et al. (2022) [164], clinical observations and case studies suggest that FMT may alleviate depressive symptoms, particularly in patients with comorbid gastrointestinal disturbances such as IBS. Reports include improvements in mood, increased appetite, reduced fatigue, and enhanced social engagement following FMT in individuals diagnosed with MDD or BD. These findings support the hypothesis that restoring gut microbial balance can beneficially modulate the microbiota–gut–brain axis, potentially influencing neurotransmitter production and systemic inflammation. Furthermore, in the context of autism, the clinical data are particularly compelling. Over 100 cases of ASD treated with FMT have been documented between 2011 and 2021. Notably, Kang et al. [165] demonstrated that approximately 50% of children with ASD experienced marked improvements in both gastrointestinal and behavioral symptoms following FMT, with some patients showing continued progress for up to two years post-treatment. More recent evidence further strengthens this perspective. According to Kwak et al. (2023) [166], FMT has been shown not only to alleviate core symptoms of ASD and associated gastrointestinal comorbidities, but also to beneficially reshape the gut microbiota composition. Specifically, it increases microbial diversity and enriches beneficial taxa such as *Bifidobacteria* and *Prevotella*, while reducing potentially pathogenic species like *Eubacterium coprostanoligenes*. These microbial shifts are believed to contribute to improved behavioral and gastrointestinal outcomes in ASD through the modulation of the microbiota–gut–brain axis. Although the application of FMT in attention-deficit/hyperactivity disorder (ADHD) remains in its early stages, preliminary findings suggest therapeutic potential in this context as well. In this study, symptomatic improvements were shown in an individual with ADHD following FMT, which were associated with an increased abundance of *Faecalibacterium prausnitzii*—a bacterium known for its anti-inflammatory properties. The authors propose that FMT may exert neuroprotective effects through the modulation of key pathways involving SCFAs, tryptophan metabolism, and the synthesis of neurotransmitters such as dopamine—mechanisms that are critically involved in ADHD pathophysiology [166]. Although studies show that FMT can modulate gut microbiota composition and improve mood-related symptoms in animal models, there is insufficient evidence to support its widespread clinical use. The challenges associated with standardizing FMT protocols, ensuring donor safety, and understanding the long-term effects of such treatments remain key obstacles in translating these findings to clinical practice [167]. The complexity of the microbiota–brain interaction calls for further research into the optimal delivery methods, treatment duration, and patient selection for FMT. In conclusion, while FMT holds substantial promise as a novel treatment for depression and other psychiatric disorders, more clinical studies are needed. The growing body of preclinical evidence, coupled with early-stage clinical trials, indicates that FMT could provide a valuable adjunct therapy for patients with TRD.

### 5.4. New Strategies for Modulating the Gut Microbiota in Mental Health

The therapeutic potential of gut microbiota modulation for treating mood disorders is a rapidly evolving field. Established approaches like prebiotics, probiotics, and FMT have already shown promising results [142,144,157]. However, the future lies in exploring next-generation strategies that target the underlying mechanisms of mood disorders with greater precision. Emerging treatments hold promise for more targeted and personalized therapies. These include postbiotics (beneficial metabolites produced by gut bacteria), specific microbial metabolites (e.g., short-chain fatty acids, neurotransmitter precursors), bacteriophage therapy, and vagus nerve stimulation [133,168]. However, challenges remain regarding regulation, safety concerns, and production difficulties [167]. Overcoming these barriers requires collaboration between scientists, industry, and regulatory bodies. Advanced technologies like gene editing and machine learning will be crucial in understanding how gut bacteria influence the brain. Recent advances in machine learning present promising tools for deciphering the intricate interplay between gut microbiota and mood disorders [169]. By analyzing metagenomic signatures, encompassing unique microbial gene fingerprints with corresponding taxonomic and metabolic data, these methodologies can facilitate the identification of robust biomarkers for targeted therapies. For example, predictive models have demonstrated the potential to differentiate SSRI responders from non-responders based on gut microbiota composition and metabolomic profiles, revealing, for instance, the upregulation of acetate degradation and neurotransmitter synthesis pathways. This supports the development of precision-based, microbiota-focused interventions. As these models mature, they may also enable earlier diagnosis and personalized, staged treatment planning [169,170]. Furthermore, investigations should explore the influences of individual factors, such as age, sex, drug use, and lifestyle, on treatment outcomes [133,171]. Sex hormones, including estrogen, progesterone, and androgens, which vary between sexes and across physiological life stages (puberty, menstrual cycle, pregnancy, and menopause), significantly modulate microbial composition and function [77]. Conversely, gut microbiota can influence the reproductive endocrine system, potentially affecting processes such as follicular development, insulin sensitivity, and inflammation. These bidirectional interactions are particularly evident in polycystic ovary syndrome (PCOS), where gut dysbiosis has been associated with hyperandrogenism, insulin resistance, and chronic low-grade inflammation, all of which may contribute to mood and cognitive disturbances [172]. Consistently, a recent case–control study revealed sex-specific gut microbiota alterations in MDD and BD. Distinct microbial signatures were observed in female patients, including an enrichment of Bacteroidaceae in MDD and Lachnospiraceae in BD [173]. These findings underscore the importance of acknowledging sex-based microbial differences.

**Figure 3:**
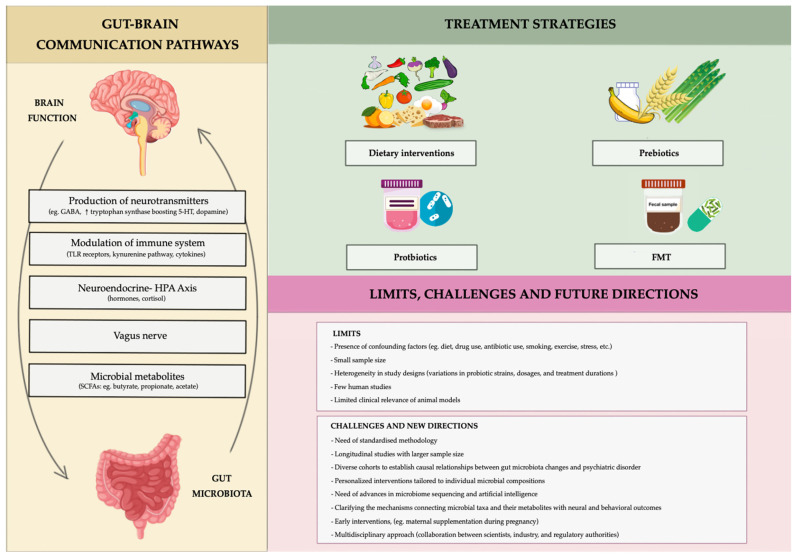
Schematic representation of gut–brain axis, along with problems and future challenges linking gut microbiota to mood disorders.

Increased funding and collaboration are essential to bring these new therapies to patients. By combining scientific knowledge with clinical practice, we can develop safe and effective treatments for mood disorders, offering new hope for those in need. Figure 3 summarizes topics illustrated so far, offering a glance at the limitations and future strategies for a better understanding of the relationship between gut microbiota and mood disorders.

## 6. Conclusions, Limitations and Future Directions

This review highlights the close relationship between gut microbiota and mental health, emphasizing the gut–brain axis as a key mediator in mood regulation. Dysbiosis, or microbial imbalance, is strongly associated with mood disorders. Thus, restoring microbial homeostasis through microbiota-targeted therapies has the potential to alleviate psychiatric symptoms, as suggested by evidence from preclinical and clinical studies. Probiotic formulations, particularly those containing *Bifidobacterium* and *Lactobacillus* species, have shown promise in modulating the gut–brain axis and reducing inflammation. Similarly, prebiotics that stimulate the growth of beneficial bacteria appear promising for improving mental health outcomes. Other interventions, such as dietary changes and FMT, are also gaining attention for their therapeutic potential. Despite some encouraging findings, significant limitations persist. Current research often relies on small sample sizes, and heterogeneity in study designs—such as variations in probiotic strains, dosages, and treatment durations—complicates the interpretation of results. Furthermore, translating discoveries from experimental models to human populations presents challenges, particularly due to environmental influences and inter-individual variability. Given the uniqueness of the gut microbiome, personalized interventions tailored to individual microbial compositions, genetic profiles, and lifestyle factors should be developed. Advances in microbiome sequencing and artificial intelligence could support these efforts. Understanding the pathways linking specific microbial taxa and their metabolites to neural and behavioral outcomes is crucial. Research should focus on how microbial metabolites, such as short-chain fatty acids and tryptophan derivatives, influence neurotransmitter systems and neuroinflammatory processes. To ensure reproducibility, standardized protocols for probiotic and prebiotic supplementation must be established. Combining microbiota-targeted approaches with established treatments, such as pharmacotherapy and psychotherapy, could enhance therapeutic outcomes. For example, probiotics could serve as adjunctive therapies to improve the efficacy of antidepressants or mitigate their side effects. Dietary interventions, including the Mediterranean diet, as well as exercise and stress reduction strategies, should be integrated into holistic treatment plans. Since the gut microbiota develops early in life and is influenced by factors such as delivery mode, breastfeeding, and antibiotic exposure, research into early interventions, including maternal supplementation during pregnancy, could help mitigate the risk of psychiatric disorders later in life.

Unlocking the therapeutic potential of the gut–brain axis requires multidisciplinary approach involving microbiology, nutrition, bioinformatics, and psychiatry. By integrating microbiota-targeted strategies into mental health care, this emerging field has the potential to revolutionize the management of mood disorders.

## Figures and Tables

**Table 1:** Microbiome alterations in bipolar disorder (BD).

Author (Year)	Changes in Microbiota
Painold et al., 2019 [75]	Decreased Abundance: *Faecalibacterium* (correlated with severe symptoms such as sleep disturbances and psychotic episodes).
Grice & Segre, 2012 [85]; Lucidi et al., 2021 [74]; Gondalia et al., 2019 [81]	Increased Abundance: *Actinobacteria* (particularly Coriobacteria), *Prevotella*, *Enterobacter* species (Gram-negative).
Lucidi et al., 2021 [74]; Gondalia et al., 2019 [81]	Increased Abundance (Gram-positive): *Atopobium Cluster*, *Clostridium*, *Flavinofractor*.
Lucidi et al., 2021 [74]	Subtype Differences: *Prevotella* more prevalent in BD type 1; *Collinsella* more abundant in BD type 2.

**Table 2:** Microbiota alterations in major depressive disorder.

Author (Year)	Microbiota Changes
Liu et al., 2016 [104]	Reduced Diversity: Decreased microbial alpha and beta diversity.
	Decreased Abundance: *Firmicutes*, *Bacteroides*, *Proteobacteria*, *Bifidobacterium*, *Lactobacillus*, *Faecalibacterium*, *Ruminococcus*.
	Increased Abundance: *Actinobacteria*, *Fusobacteria*, *Prevotellaceae*, *Lachnospiraceae*, *Flavonifractor*.
Aizawa et al., 2016 [105]	Depletion: *Coprococcus*, *Dialister*; associations noted with *Alistipes*, *Faecalibacterium*, and *Ruminococcus*.

**Table 3:** Gut microbiota alterations and their links to mood disorders via dietary and prebiotic interventions.

Therapeutic Approach	Main Effect on Gut Microbiota	Type of Article	Author (Year)
Dietary Modulation (e.g., Mediterranean Diet)	Promotes gut microbial diversity and increases beneficial bacteria, such as those producing SCFAs. Reduces inflammation and improves brain function. Associated with better mental outcome.	Review	Ribeiro et al., 2022 [126]
		Systematic review	Swainson et al., 2023 [127]
Flavonoid-Rich Foods (e.g., Orange Juice)	Increases *Lachnospiraceae* and *Bifidobacterium* abundance, correlated with serum BDNF levels; reduced *Clostridium*, potentially improving depressive symptoms.	Randomized Controlled Trial	Park et al., 2020 [128]
Prebiotic Supplementation (e.g., fructans, GOS)	Enhances beneficial bacteria (e.g., *Bacteroides*, *Parabacteroides*); may support mental health indirectly by improving gut microbiota composition.	Clinical study	Thompson et al., 2024 [129]
24-week 4G-beta-D-Galactosylsucrose (LS) supplementation	Increases microbial diversity and *Bifidobacterium* abundance. Limited direct improvement in depressive symptoms.	Randomized Controlled Trial	Tarutani et al., 2022 [130]
8-week inulin supplementation	No significant impact on gut microbiota composition or function.	Randomized Controlled Trial	Vaghef-Mehrabani et al., 2023 [131]

Abbreviations: GOS—galactooligosaccharides; BDNF—brain-derived neurotrophic factor; SCFAs—short-chain fatty acids.

**Table 4:** Recent clinical trials and meta-analyses investigating the effects of psychobiotics in mood disorders.

Author (Year)	Study Design	Study Sample	Duration (Weeks)	Probiotic Characteristics	Results
Xiang Ng et al. (2018) [144]	Meta-analysis	1349 Participants (1147 healthy individuals; 40 MDD; 44 with IBS and mild to moderate anxiety and/or depression; 39 with CFS; 79 with at least moderate scores on self-report mood measures)	-	Various probiotic strains, doses, and durations	No significant impact on mood overall, but significant benefit for mild to moderate depression (SMD = −0.684, *p* = 0.029). Adjunctive therapy potential.
Tian et al. (2022) [145]	Placebo-controlled, double-blind RCT	45 MDD patients	4	*Bifidobacterium breve* CCFM1025 (10^10^ CFU daily)	Significant reductions in depressive symptoms (HDRS-24 *p* = 0.036; MADRS *p* = 0.037), changes in tryptophan metabolism and serotonin turnover; mild changes in gut microbial composition.
Schneider et al. (2022) [146]	Placebo-controlled, double-blind RCT	60 MDD patients	4	Multi-strain probiotic (900 billion CFU daily, including *Bifidobacterium* spp., *Lactobacillus* spp., *S. thermophilus*)	Improved immediate recall on VLMT, reduced hippocampal activation, no significant changes in BDNF levels or other cognitive measures.
Yamanbaeva et al. (2022) [147]	Placebo-controlled, double-blind RCT	32 MDD patients	4	Multi-strain probiotic (e.g., *Bifidobacterium* spp., *Lactobacillus* spp., *S. thermophilus*)	Stabilized uncinate fasciculus diffusivity, improved resting-state connectivity, reduced hippocampal activation, cognitive and emotional improvements.
Zhang et al. (2022) [148]	Placebo-controlled, double-blind RCT	82 depressed patients with constipation	9	*Lacticaseibacillus paracasei* strain Shirota (LcS, 10^8 CFU/mL)	Improved specific constipation symptoms, beneficial gut microbiota changes, reduced IL-6. No significant differences in depressive symptoms vs. placebo.
Casertano et al. (2024) [149]	Randomized, double-blind, placebo-controlled, cross-over study	77 healthy adults, with mild/moderate stress score in the DASS-42 questionnaire	12	*Levilactobacillus brevis* P30021, *Lactiplantibacillus plantarum* P30025	Reduced rumination (LEIDS-r), no significant effects on cognitive performance (DASS-42), increased probiotic genera abundance.
Chahwan et al. (2019) [150]	Placebo-controlled, triple-blind, RCT.	71 depressed patients	8	Multi-strain probiotic (e.g., *Bifidobacterium* spp., *Lactobacillus* spp., *Lactococcus lactis*)	Reduced cognitive reactivity, shifted participants from clinical to no depression diagnosis, no significant gut microbiota changes.
Rudzki et al. (2024) [151]	Placebo-controlled, double-blind RCT	79 MDD patients on SSRI	8	*Lactiplantibacillus plantarum* 299 v (10 × 10^9^ CFU per capsule)	Improved cognitive function, decreased kynurenine levels, increased the 3-HK/KA ratio. Cognitive performance and mood regulation improvements.
Reininghaus et al. (2018) [100]	Pilot study	20 euthymic BD patients	12	Multi-strain probiotic (e.g., *Bifidobacterium* spp., *Lactobacillus* spp., *Lactococcus lactis*)	Improved attention, psychomotor speed, and executive function. Highlights cognitive benefits for BD.
Borkent et al. (2024) [152]	Double-blind, placebo-controlled RCT	BD and SSD patients	12	Multi-strain probiotic (e.g., *Bifidobacterium* spp., *Lactobacillus* spp., *Lactococcus lactis*)	Borderline improvement in verbal memory, positive effects on intestinal permeability, inflammation markers reduced (zonulin, alpha-1 antitrypsin).

Abbreviations: 3-HK/KA, 3-hydroxykynurenine:kynurenine; BD, bipolar disorder; BDNF, brain-derived neurotrophic factor; CFS, chronic fatigue syndrome; CFU, colony forming unit; DASS-42, Depression Anxiety and Stress Scale (-42); HDRS, Hamilton Depression Rating Scale; MADRS, Montgomery–Asberg Depression Rating Scale; LEIDS-r, Leiden Index of Depression Sensitivity-Revised; MDD, major depressive disorder; RCT, randomized controlled trial; SMD, standardized mean difference; SSD, schizophrenia spectrum disorder; VLMT, Verbal Learning Memory Test.

## References

[1] Thursby E., Juge N. (2017). Introduction to the human gut microbiota. Biochem. J..

[2] Cresci G.A., Bawden E. (2015). Gut Microbiome. Nutr. Clin. Pract..

[3] Yao S., Zhao Z., Wang W., Liu X. (2021). Bifidobacterium Longum: Protection against Inflammatory Bowel Disease. J. Immunol. Res..

[4] Fernández J., Saettone P., Franchini M.C., Villar C.J., Lombó F. (2022). Antitumor bioactivity and gut microbiota modulation of polyhydroxybutyrate (PHB) in a rat animal model for colorectal cancer. Int. J. Biol. Macromol..

[5] Toader C., Dobrin N., Costea D., Glavan L.A., Covache-Busuioc R.A., Dumitrascu D.I., Bratu B.G., Costin H.P., Ciurea A.V. (2024). Mind, Mood and Microbiota-Gut-Brain Axis in Psychiatric Disorders. Int. J. Mol. Sci..

[6] Chen M., Ruan G., Chen L., Ying S., Li G., Xu F., Xiao Z., Tian Y., Lv L., Ping Y. (2022). Neurotransmitter and Intestinal Interactions: Focus on the Microbiota-Gut-Brain Axis in Irritable Bowel Syndrome. Front. Endocrinol..

[7] Vuong H.E., Hsiao E.Y. (2017). Emerging Roles for the Gut Microbiome in Autism Spectrum Disorder. Biol. Psychiatry.

[8] James S.L. (2018). Global, regional, and national incidence, prevalence, and years lived with disability for 354 diseases and injuries for 195 countries and territories, 1990–2017: A systematic analysis for the Global Burden of Disease Study 2017. Lancet.

[9] Cryan J.F., Dinan T.G. (2012). Mind-altering microorganisms: The impact of the gut microbiota on brain and behaviour. Nat. Rev. Neurosci..

[10] Evans S.J., Bassis C.M., Hein R., Assari S., Flowers S.A., Kelly M.B., Young V.B., Ellingrod V.E., McInnis M.G. (2017). The gut microbiome composition associates with bipolar disorder and illness severity. J. Psychiatr. Res..

[11] Zheng P., Yang J., Li Y., Wu J., Liang W., Yin B., Tan X., Huang Y., Chai T., Zhang H. (2020). Gut Microbial Signatures Can Discriminate Unipolar from Bipolar Depression. Adv. Sci..

[12] Lin X., Huang J., Wang S., Zhang K. (2024). Bipolar disorder and the gut microbiota: A bibliometric analysis. Front. Neurosci..

[13] Li Z., Lai J., Zhang P., Ding J., Jiang J., Liu C., Huang H., Zhen H., Xi C., Sun Y. (2022). Multi-omics analyses of serum metabolome, gut microbiome and brain function reveal dysregulated microbiota-gut-brain axis in bipolar depression. Mol. Psychiatry.

[14] Valle C.G.-D., Fernández J., Solá E., Montoya-Castilla I., Morillas C., Bañuls C. (2023). Association between gut microbiota and psychiatric disorders: A systematic review. Front. Pshychology.

[15] Clapp M., Aurora N., Herrera L., Bhatia M., Wilen E., Wakefield S. (2017). Gut Microbiota’s Effect on Mental Health: The Gut-Brain Axis. Clin. Pract..

[16] Merlo G., Bachtel G., Sugden S.G. (2024). Gut microbiota, nutrition, and mental health. Front. Nutr..

[17] Xiong R.G., Li J., Cheng J., Zhou D.D., Wu S.X., Huang S.Y., Saimaiti A., Yang Z.J., Gan R.Y., Li H.B. (2023). The Role of Gut Microbiota in Anxiety, Depression, and Other Mental Disorders as Well as the Protective Effects of Dietary Components. Nutrients.

[18] Gomaa E.Z. (2020). Human gut microbiota/microbiome in health and diseases: A review. Antonie van Leeuwenhoek.

[19] García-Montero C., Fraile-Martínez O., Gómez-Lahoz A.M., Pekarek L., Castellanos A.J., Noguerales-Fraguas F., Coca S., Guijarro L.G., García-Honduvilla N., Asúnsolo A. (2021). Nutritional components in western diet versus mediterranean diet at the gut microbiota-immune system interplay. implications for health and disease. Nutrients.

[20] Rinninella E., Raoul P., Cintoni M., Franceschi F., Miggiano G.A.D., Gasbarrini A., Mele M.C. (2019). What is the healthy gut microbiota composition? A changing ecosystem across age, environment, diet, and diseases. Microorganisms.

[21] Van Hul M., Cani P.D., Petitfils C., De Vos W.M., Tilg H., El-Omar E.M. (2024). What defines a healthy gut microbiome?. Gut.

[22] Hou K., Wu Z.X., Chen X.Y. (2022). Microbiota in health and diseases. Signal Transduct. Target Ther..

[23] Berding K., Vlckova K., Marx W., Schellekens H., Stanton C., Clarke G., Jacka F., Dinan T.G., Cryan J.F. (2021). Diet and the Microbiota-Gut-Brain Axis: Sowing the Seeds of Good Mental Health. Adv. Nutr..

[24] Finotello F., Mastrorilli E., Di Camillo B. (2018). Measuring the diversity of the human microbiota with targeted next-generation sequencing. Brief. Bioinform..

[25] Marano G., Mazza M., Lisci F.M., Ciliberto M., Traversi G., Kotzalidis G.D., De Berardis D., Laterza L., Sani G., Gasbarrini A. (2023). The Microbiota–Gut–Brain Axis: Psychoneuroimmunological Insights. Nutrients.

[26] Adak A., Khan M.R. (2019). An insight into gut microbiota and its functionalities. Cell. Mol. Life Sci..

[27] Meerveld B.G.-V., Johnson A.C., Grundy D. (2017). Gastrointestinal Physiology and Function. Gastrointestinal Pharmacology.

[28] Koga Y. (2022). Microbiota in the stomach and application of probiotics to gastroduodenal diseases. World J. Gastroenterol..

[29] Ohno H., Satoh-Takayama N. (2020). Stomach microbiota, *Helicobacter pylori*, and group 2 innate lymphoid cells. Exp. Mol. Med..

[30] Procházková N., Falony G., Dragsted L.O., Licht T.R., Raes J., Roager H.M. (2023). Advancing human gut microbiota research by considering gut transit time. Gut.

[31] Sun Y., Zhang S., Nie Q., He H., Tan H., Geng F., Ji H., Hu J., Nie S. (2023). Gut firmicutes: Relationship with dietary fiber and role in host homeostasis. Crit. Rev. Food Sci. Nutr..

[32] Wang L.Y., He L.H., Xu L.J., Li S.B. (2024). Short-chain fatty acids: Bridges between diet, gut microbiota, and health. J. Gastroenterol. Hepatol..

[33] Cataldi S., Poli L., Şahin F.N., Patti A., Santacroce L., Bianco A., Greco G., Ghinassi B., Di Baldassarre A., Fischetti F. (2022). The Effects of Physical Activity on the Gut Microbiota and the Gut–Brain Axis in Preclinical and Human Models: A Narrative Review. Nutrients.

[34] Vandenplas Y., Carnielli V.P., Ksiazyk J., Luna M.S., Migacheva N., Mosselmans J.M., Picaud J.C., Possner M., Singhal A., Wabitsch M. (2020). Factors affecting early-life intestinal microbiota development. Nutrition.

[35] Zhang C., Li L., Jin B., Xu X., Zuo X., Li Y., Li Z. (2021). The Effects of Delivery Mode on the Gut Microbiota and Health: State of Art. Front. Microbiol..

[36] Suárez-Martínez C., Santaella-Pascual M., Yagüe-Guirao G., Martínez-Graciá C. (2023). Infant gut microbiota colonization: Influence of prenatal and postnatal factors, focusing on diet. Front. Microbiol..

[37] Chong H.-Y., Tan L.T., Law J.W., Hong K.W., Ratnasingam V., Ab Mutalib N.S., Lee L.H., Letchumanan V. (2022). Exploring the Potential of Human Milk and Formula Milk on Infants’ Gut and Health. Nutrients.

[38] Yaron S., Shachar D., Abramas L., Riskin A., Bader D., Litmanovitz I., Bar-Yoseph F., Cohen T., Levi L., Lifshitz Y. (2013). Effect of High β-Palmitate Content in Infant Formula on the Intestinal Microbiota of Term Infants. J. Pediatr. Gastroenterol. Nutr..

[39] Van den Elsen L.W.J., Garssen J., Burcelin R., Verhasselt V. (2019). Shaping the Gut Microbiota by Breastfeeding: The Gateway to Allergy Prevention?. Front. Pediatr..

[40] Mangiola F., Nicoletti A., Gasbarrini A., Ponziani F.R. (2018). Gut microbiota and aging. Eur. Rev. Med. Pharmacol. Sci..

[41] Du Y., Gao X.-R., Peng L., Ge J.-F. (2020). Crosstalk between the microbiota-gut-brain axis and depression. Heliyon.

[42] Sekirov I., Russell S.L., Antunes L.C.M., Finlay B.B. (2010). Gut Microbiota in Health and Disease. Physiol. Rev..

[43] Foster J.A., Neufeld K.A.M. (2013). Gut-brain axis: How the microbiome influences anxiety and depression. Trends Neurosci..

[44] Karlsson F.H., Ussery D.W., Nielsen J., Nookaew I. (2011). A Closer Look at Bacteroides: Phylogenetic Relationship and Genomic Implications of a Life in the Human Gut. Microb. Ecol..

[45] Severance E., Tveiten D., Lindström L., Yolken R., Reichelt K. (2016). The Gut Microbiota and the Emergence of Autoimmunity: Relevance to Major Psychiatric Disorders. Curr. Pharm. Des..

[46] Ismail A.S., Hooper L.V. (2005). Epithelial Cells and Their Neighbors. IV. Bacterial contributions to intestinal epithelial barrier integrity. Am. J. Physiol. Gastrointest. Liver Physiol..

[47] Yatsunenko T., Rey F.E., Manary M.J., Trehan I., Dominguez-Bello M.G., Contreras M., Magris M., Hidalgo G., Baldassano R.N., Anokhin A.P. (2012). Human gut microbiome viewed across age and geography. Nature.

[48] Davey K.J., Cotter P.D., O’Sullivan O., Crispie F., Dinan T.G., Cryan J.F., O’Mahony S.M. (2013). Antipsychotics and the gut microbiome: Olanzapine-induced metabolic dysfunction is attenuated by antibiotic administration in the rat. Transl. Psychiatry.

[49] Dominguez-Bello M.G., Costello E.K., Contreras M., Magris M., Hidalgo G., Fierer N., Knight R. (2010). Delivery mode shapes the acquisition and structure of the initial microbiota across multiple body habitats in newborns. Proc. Natl. Acad. Sci. USA.

[50] Fallani M., Young D., Scott J., Norin E., Amarri S., Adam R., Aguilera M., Khanna S., Gil A., Edwards C.A. (2010). Intestinal microbiota of 6-week-old infants across Europe: Geographic influence beyond delivery mode, breast-feeding, and antibiotics. J. Pediatr. Gastroenterol. Nutr..

[51] Patterson E., Ryan P.M., Wiley N., Carafa I., Sherwin E., Moloney G., Franciosi E., Mandal R., Wishart D.S., Tuohy K. (2019). Gamma-aminobutyric acid-producing lactobacilli positively affect metabolism and depressive-like behaviour in a mouse model of metabolic syndrome. Sci. Rep..

[52] Busnelli M., Manzini S., Chiesa G. (2020). The gut microbiota affects host pathophysiology as an endocrine organ: A focus on cardiovascular disease. Nutrients.

[53] Fülling C., Dinan T.G., Cryan J.F. (2019). Gut Microbe to Brain Signaling: What Happens in Vagus. Neuron.

[54] Wang Y., Zhan G., Cai Z., Jiao B., Zhao Y., Li S., Luo A. (2021). Vagus nerve stimulation in brain diseases: Therapeutic applications and biological mechanisms. Neurosci. Biobehav. Rev..

[55] Bonaz B., Bazin T., Pellissier S. (2018). The Vagus Nerve at the Interface of the Microbiota-Gut-Brain Axis. Front. Neurosci..

[56] Wang G.-J. (2018). Food addiction A common neurobiological mechanism with drug abuse. Front. Biosci..

[57] Wang Y., Duan C., Du X., Zhu Y., Wang L., Hu J., Sun Y. (2024). Vagus Nerve and Gut-Brain Communication. Neuroscientist.

[58] Scaldaferri F., Gerardi V., Lopetuso L.R., Del Zompo F., Mangiola F., Boškoski I., Bruno G., Petito V., Laterza L., Cammarota G. (2013). Gut Microbial Flora, Prebiotics, and Probiotics in IBD: Their Current Usage and Utility. Biomed. Res. Int..

[59] Bolon B. (2012). Cellular and Molecular Mechanisms of Autoimmune Disease. Toxicol. Pathol..

[60] Dinan T.G., Cryan J.F. (2015). The impact of gut microbiota on brain and behaviour. Curr. Opin. Clin. Nutr. Metab. Care.

[61] Round J.L., O’Connell R.M., Mazmanian S.K. (2010). Coordination of tolerogenic immune responses by the commensal microbiota. J. Autoimmun..

[62] Misiak B., Łoniewski I., Marlicz W., Frydecka D., Szulc A., Rudzki L., Samochowiec J. (2020). The HPA axis dysregulation in severe mental illness: Can we shift the blame to gut microbiota?. Prog. Neuropsychopharmacol. Biol. Psychiatry.

[63] Ma Q., Xing C., Long W., Wang H.Y., Liu Q., Wang R.-F. (2019). Impact of microbiota on central nervous system and neurological diseases: The gut-brain axis. J. Neuroinflammation.

[64] Doroszkiewicz J., Groblewska M., Mroczko B. (2021). The Role of Gut Microbiota and Gut–Brain Interplay in Selected Diseases of the Central Nervous System. Int. J. Mol. Sci..

[65] Clarke G., Stone T.W., Schwarcz R. (2017). The kynurenine pathway: Towards metabolic equilibrium. Neuropharmacology.

[66] Schwarcz R., Stone T.W. (2017). The kynurenine pathway and the brain: Challenges, controversies and promises. Neuropharmacology.

[67] Kadriu B., Farmer C.A., Yuan P., Park L.T., Deng Z.D., Moaddel R., Henter I.D., Shovestul B., Ballard E.D., Kraus C. (2021). The kynurenine pathway and bipolar disorder: Intersection of the monoaminergic and glutamatergic systems and immune response. Mol. Psychiatry.

[68] Potter M.C., Elmer G.I., Bergeron R., Albuquerque E.X., Guidetti P., Wu H.Q., Schwarcz R. (2010). Reduction of Endogenous Kynurenic Acid Formation Enhances Extracellular Glutamate, Hippocampal Plasticity, and Cognitive Behavior. Neuropsychopharmacology.

[69] Lin P., Li D., Shi Y., Li Q., Guo X., Dong K., Chen Q., Lou X., Li Z., Li P. (2023). Dysbiosis of the Gut Microbiota and Kynurenine (Kyn) Pathway Activity as Potential Biomarkers in Patients with Major Depressive Disorder. Nutrients.

[70] Inam M.E., Inam M.E., Enduru N., Quevedo J., Zhao Z. (2023). The kynurenine pathway in major depressive disorder, bipolar disorder, and schizophrenia: A systematic review and meta-analysis of cerebrospinal fluid studies. Braz. J. Psychiatry.

[71] Nettis M.A., Lombardo G., Hastings C., Zajkowska Z., Mariani N., Nikkheslat N., Sforzini L., Worrell C., Begum A., Brown M. (2023). The interaction between kynurenine pathway, suicidal ideation and augmentation therapy with minocycline in patients with treatment-resistant depression. J. Psychopharmacol..

[72] Dash S., Clarke G., Berk M., Jacka F.N. (2015). The gut microbiome and diet in psychiatry. Curr. Opin. Psychiatry.

[73] Lach G., Schellekens H., Dinan T.G., Cryan J.F. (2018). Anxiety, Depression, and the Microbiome: A Role for Gut Peptides. Neurotherapeutics.

[74] Lucidi L., Pettorruso M., Vellante F., Di Carlo F., Ceci F., Santovito M.C., Di Muzio I., Fornaro M., Ventriglio A., Tomasetti C. (2021). Gut Microbiota and Bipolar Disorder: An Overview on a Novel Biomarker for Diagnosis and Treatment. Int. J. Mol. Sci..

[75] Painold A., Mörkl S., Kashofer K., Halwachs B., Dalkner N., Bengesser S., Birner A., Fellendorf F., Platzer M., Queissner R. (2019). A step ahead: Exploring the gut microbiota in inpatients with bipolar disorder during a depressive episode. Bipolar Disord..

[76] Jiang H., Ling Z., Zhang Y., Mao H., Ma Z., Yin Y., Wang W., Tang W., Tan Z., Shi J. (2015). Altered fecal microbiota composition in patients with major depressive disorder. Brain Behav. Immun..

[77] Marano G., Traversi G., Gaetani E., Gasbarrini A., Mazza M. (2023). Gut microbiota in women: The secret of psychological and physical well-being. World J. Gastroenterol..

[78] Naseribafrouei A., Hestad K., Avershina E., Sekelja M., Linløkken A., Wilson R., Rudi K. (2014). Correlation between the human fecal microbiota and depression. Neurogastroenterol. Motil..

[79] Huang T.-T., Lai J.-B., Du Y.-L., Xu Y., Ruan L.-M., Hu S.-H. (2019). Current Understanding of Gut Microbiota in Mood Disorders: An Update of Human Studies. Front. Genet..

[80] Huang Y., Shi X., Li Z., Shen Y., Shi X., Wang L., Li G., Yuan Y., Wang J., Zhang Y. (2018). Possible association of Firmicutes in the gut microbiota of patients with major depressive disorder. Neuropsychiatr. Dis. Treat..

[81] Gondalia S., Parkinson L., Stough C., Scholey A. (2019). Gut microbiota and bipolar disorder: A review of mechanisms and potential targets for adjunctive therapy. Psychopharmacology.

[82] Borodovitsyna O., Flamini M., Chandler D. (2017). Noradrenergic Modulation of Cognition in Health and Disease. Neural. Plast..

[83] Klein M.O., Battagello D.S., Cardoso A.R., Hauser D.N., Bittencourt J.C., Correa R.G. (2019). Dopamine: Functions, Signaling, and Association with Neurological Diseases. Cell. Mol. Neurobiol..

[84] Kleinridders A., Pothos E.N. (2019). Impact of Brain Insulin Signaling on Dopamine Function, Food Intake, Reward, and Emotional Behavior. Curr. Nutr. Rep..

[85] Grice E.A., Segre J.A. (2012). The Human Microbiome: Our Second Genome. Annu. Rev. Genomics Hum. Genet..

[86] Zhang P., Kong L., Huang H., Pan Y., Zhang D., Jiang J., Shen Y., Xi C., Lai J., Ng C.H. (2022). Gut Microbiota—A Potential Contributor in the Pathogenesis of Bipolar Disorder. Front. Neurosci..

[87] Rieder R., Wisniewski P.J., Alderman B.L., Campbell S.C. (2017). Microbes and mental health: A review. Brain Behav. Immun..

[88] Cryan J.F., O’Riordan K.J., Cowan C.S.M., Sandhu K.V., Bastiaanssen T.F.S., Boehme M., Codagnone M.G., Cussotto S., Fulling C., Golubeva A.V. (2019). The Microbiota-Gut-Brain Axis. Physiol. Rev..

[89] Caso J., Balanzá-Martínez V., Palomo T., García-Bueno B. (2016). The Microbiota and Gut-Brain Axis: Contributions to the Immunopathogenesis of Schizophrenia. Curr. Pharm. Des..

[90] Addolorato G., De Lorenzi G., Abenavoli L., Leggio L., Capristo E., Gasbarrini G. (2004). Psychological support counselling improves gluten-free diet compliance in coeliac patients with affective disorders. Aliment. Pharmacol. Ther..

[91] Fond G., Loundou A., Hamdani N., Boukouaci W., Dargel A., Oliveira J., Roger M., Tamouza R., Leboyer M., Boyer L. (2014). Anxiety and depression comorbidities in irritable bowel syndrome (IBS): A systematic review and meta-analysis. Eur. Arch. Psychiatry Clin. Neurosci..

[92] Gao J. (2013). Correlation between anxiety-depression status and cytokines in diarrhea-predominant irritable bowel syndrome. Exp. Ther. Med..

[93] Bai Y.-M., Su T.P., Tsai S.J., Wen-Fei C., Li C.T., Pei-Chi T., Mu-Hong C. (2014). Comparison of inflammatory cytokine levels among type I/type II and manic/hypomanic/euthymic/depressive states of bipolar disorder. J. Affect. Disord..

[94] Anderson G., Maes M. (2015). Bipolar Disorder: Role of Immune-Inflammatory Cytokines, Oxidative and Nitrosative Stress and Tryptophan Catabolites. Curr. Psychiatry Rep..

[95] Flowers S.A., Ward K.M., Clark C.T. (2020). The gut microbiome in bipolar disorder and pharmacotherapy management. Neuropsychobiology.

[96] Marano G., Traversi G., Gaetani E., Pola R., Claro A.E., Mazza M. (2022). Alcohol use disorder and liver injury related to the COVID-19 pandemic. World J. Hepatol..

[97] Neuman M.G., French S.W., Zakhari S., Malnick S., Seitz H.K., Cohen L.B., Salaspuro M., Voinea-Griffin A., Barasch A., Kirpich I.A. (2017). Alcohol, microbiome, life style influence alcohol and non-alcoholic organ damage. Exp. Mol. Pathol..

[98] Legendre T., Boudebesse C., Henry C., Etain B. (2017). Antibiomania: Penser au syndrome maniaque secondaire à une antibiothérapie. Encephale.

[99] Kelly J.R., Kennedy P.J., Cryan J.F., Dinan T.G., Clarke G., Hyland N.P. (2015). Breaking down the barriers: The gut microbiome, intestinal permeability and stress-related psychiatric disorders. Front. Cell. Neurosci..

[100] Reininghaus E.Z., Wetzlmair L.C., Fellendorf F.T., Platzer M., Queissner R., Birner A., Pilz R., Hamm C., Maget A., Koidl C. (2020). The Impact of Probiotic Supplements on Cognitive Parameters in Euthymic Individuals with Bipolar Disorder: A Pilot Study. Neuropsychobiology.

[101] Winter G., Hart R.A., Charlesworth R.P.G., Sharpley C.F. (2018). Gut microbiome and depression: What we know and what we need to know. Rev. Neurosci..

[102] Li N., Wang Q., Wang Y., Sun A., Lin Y., Jin Y., Li X. (2019). Fecal microbiota transplantation from chronic unpredictable mild stress mice donors affects anxiety-like and depression-like behavior in recipient mice via the gut microbiota-inflammation-brain axis. Stress.

[103] Simpson C.A., Diaz-Arteche C., Eliby D., Schwartz O.S., Simmons J.G., Cowan C.S.M. (2021). The gut microbiota in anxiety and depression—A systematic review. Clin. Psychol. Rev..

[104] Liu Y., Zhang L., Wang X., Wang Z., Zhang J., Jiang R., Wang X., Wang K., Liu Z., Xia Z. (2016). Similar Fecal Microbiota Signatures in Patients with Diarrhea-Predominant Irritable Bowel Syndrome and Patients with Depression. Clin. Gastroenterol. Hepatol..

[105] Aizawa E., Tsuji H., Asahara T., Takahashi T., Teraishi T., Yoshida S., Ota M., Koga N., Hattori K., Kunugi H. (2016). Possible association of Bifidobacterium and Lactobacillus in the gut microbiota of patients with major depressive disorder. J. Affect. Disord..

[106] David L.A., Maurice C.F., Carmody R.N., Gootenberg D.B., Button J.E., Wolfe B.E., Ling A.V., Devlin A.S., Varma Y., Fischbach M.A. (2014). Diet rapidly and reproducibly alters the human gut microbiome. Nature.

[107] He Y., He Y., Wu W., Zheng H.M., Li P., McDonald D., Sheng H.F., Chen M.X., Chen Z.H., Ji G.Y. (2018). Regional variation limits applications of healthy gut microbiome reference ranges and disease models. Nat. Med..

[108] Bonder M.J., Kurilshikov A., Tigchelaar E.F., Mujagic Z., Imhann F., Vila A.V., Deelen P., Vatanen T., Schirmer M., Smeekens S.P. (2016). The effect of host genetics on the gut microbiome. Nat. Genet..

[109] O’Toole P.W., Jeffery I.B. (2015). Gut microbiota and aging. Science.

[110] Zhao L., Zheng S., Su G., Lu X., Yang J., Xiong Z., Wu C. (2015). In vivo study on the neurotransmitters and their metabolites change in depressive disorder rat plasma by ultra high performance liquid chromatography coupled to tandem mass spectrometry. J. Chromatogr. B.

[111] Yadid G., Friedman A. (2008). Dynamics of the dopaminergic system as a key component to the understanding of depression. Serotonin–Dopamine Interaction: Experimental Evidence and Therapeutic Relevance.

[112] Willner P., Hale A.S., Argyropoulos S. (2005). Dopaminergic mechanism of antidepressant action in depressed patients. J. Affect. Disord..

[113] Fidalgo T.M., Morales-Quezada J.L., Muzy G.S., Chiavetta N.M., Mendonca M.E., Santana M.V., Goncalves O.F., Brunoni A.R., Fregni F. (2014). Biological Markers in Noninvasive Brain Stimulation Trials in Major Depressive Disorder. J. ECT.

[114] Sudo N., Chida Y., Aiba Y., Sonoda J., Oyama N., Yu X.N., Kubo C., Koga Y. (2004). Postnatal microbial colonization programs the hypothalamic–pituitary–adrenal system for stress response in mice. J. Physiol..

[115] Rogers G.B., Keating D.J., Young R.L., Wong M.-L., Licinio J., Wesselingh S. (2016). From gut dysbiosis to altered brain function and mental illness: Mechanisms and pathways. Mol. Psychiatry.

[116] Bravo J.A., Forsythe P., Chew M.V., Escaravage E., Savignac H.M., Dinan T.G., Bienenstock J., Cryan J.F. (2011). Ingestion of *Lactobacillus* strain regulates emotional behavior and central GABA receptor expression in a mouse via the vagus nerve. Proc. Natl. Acad. Sci. USA.

[117] Han W., Tellez L.A., Perkins M.H., Perez I.O., Qu T., Ferreira J., Ferreira T.L., Quinn D., Liu Z.W., Gao X.B. (2018). A Neural Circuit for Gut-Induced Reward. Cell.

[118] Bansal T., Englert D., Lee J., Hegde M., Wood T.K., Jayaraman A. (2007). Differential Effects of Epinephrine, Norepinephrine, and Indole on *Escherichia coli* O157:H7 Chemotaxis, Colonization, and Gene Expression. Infect. Immun..

[119] O’Donnell P.M., Aviles H., Lyte M., Sonnenfeld G. (2006). Enhancement of In Vitro Growth of Pathogenic Bacteria by Norepinephrine: Importance of Inoculum Density and Role of Transferrin. Appl. Environ. Microbiol..

[120] Hoban A.E., Moloney R.D., Golubeva A.V., McVey Neufeld K.A., O’Sullivan O., Patterson E., Stanton C., Dinan T.G., Clarke G., Cryan J.F. (2016). Behavioural and neurochemical consequences of chronic gut microbiota depletion during adulthood in the rat. Neuroscience.

[121] Liao J.F., Hsu C.C., Chou G.T., Hsu J.S., Liong M.T., Tsai Y.C. (2019). Lactobacillus paracasei PS23 reduced early-life stress abnormalities in maternal separation mouse model. Benef. Microbes.

[122] Wei C.-L., Wang S., Yen J.T., Cheng Y.F., Liao C.L., Hsu C.C., Wu C.C., Tsai Y.C. (2019). Antidepressant-like activities of live and heat-killed Lactobacillus paracasei PS23 in chronic corticosterone-treated mice and possible mechanisms. Brain Res..

[123] Koronyo-Hamaoui M., Ko M.K., Koronyo Y., Azoulay D., Seksenyan A., Kunis G., Pham K., Bakhsheshian J., Rogeri P., Black K.L. (2009). Attenuation of AD-like neuropathology by harnessing peripheral immune cells: Local elevation of IL-10 and MMP-9. J. Neurochem..

[124] Dinan T.G., Stilling R.M., Stanton C., Cryan J.F. (2015). Collective unconscious: How gut microbes shape human behavior. J. Psychiatr. Res..

[125] Marx W., Lane M., Hockey M., Aslam H., Berk M., Walder K., Borsini A., Firth J., Pariante C.M., Berding K. (2021). Diet and depression: Exploring the biological mechanisms of action. Mol. Psychiatry.

[126] Ribeiro G., Ferri A., Clarke G., Cryan J.F. (2022). Diet and the microbiota-gut-brain-axis: A primer for clinical nutrition. Curr. Opin. Clin. Nutr. Metab. Care.

[127] Swainson J., Reeson M., Malik U., Stefanuk I., Cummins M., Sivapalan S. (2023). Diet and depression: A systematic review of whole dietary interventions as treatment in patients with depression. J. Affect. Disord..

[128] Park M., Choi J., Lee H.-J. (2020). Flavonoid-Rich Orange Juice Intake and Altered Gut Microbiome in Young Adults with Depressive Symptom: A Randomized Controlled Study. Nutrients.

[129] Thompson R.S., Bowers S.J., Vargas F., Hopkins S., Kelley T., Gonzalez A., Lowry C.A., Dorrestein P.C., Vitaterna M.H., Turek F.W. (2024). A Prebiotic Diet Containing Galactooligosaccharides and Polydextrose Produces Dynamic and Reproducible Changes in the Gut Microbial Ecosystem in Male Rats. Nutrients.

[130] Tarutani S., Omori M., Ido Y., Yano M., Komatsu T., Okamura T. (2022). Effects of 4G-beta-D-Galactosylsucrose in patients with depression: A randomized, double-blinded, placebo-controlled, parallel-group comparative study. J. Psychiatr. Res..

[131] Vaghef-Mehrabani E., Harouni R., Behrooz M., Ranjbar F., Asghari-Jafarabadi M., Ebrahimi-Mameghani M. (2023). Effects of inulin supplementation on inflammatory biomarkers and clinical symptoms of women with obesity and depression on a calorie-restricted diet: A randomised controlled clinical trial. Br. J. Nutr..

[132] Davani-Davari D., Negahdaripour M., Karimzadeh I., Seifan M., Mohkam M., Masoumi S.J., Berenjian A., Ghasemi Y. (2019). Prebiotics: Definition, Types, Sources, Mechanisms, and Clinical Applications. Foods.

[133] Yadav M.K., Kumari I., Singh B., Sharma K.K., Tiwari S.K. (2022). Probiotics, prebiotics and synbiotics: Safe options for next-generation therapeutics. Appl. Microbiol. Biotechnol..

[134] Vandeputte D., Falony G., Vieira-Silva S., Wang J., Sailer M., Theis S., Verbeke K., Raes J. (2017). Prebiotic inulin-type fructans induce specific changes in the human gut microbiota. Gut.

[135] Ribera C., Sánchez-Ortí J.V., Clarke G., Marx W., Mörkl S., Balanzá-Martínez V. (2024). Probiotic, prebiotic, synbiotic and fermented food supplementation in psychiatric disorders: A systematic review of clinical trials. Neurosci. Biobehav. Rev..

[136] Hill C., Guarner F., Reid G. (2014). The International Scientific Association for Probiotics and Prebiotics consensus statement on the scope and appropriate use of the term probiotic. Nat. Rev. Gastroenterol. Hepatol..

[137] Binda S., Tremblay A., Iqbal U.H., Kassem O., Le Barz M., Thomas V., Bronner S., Perrot T., Ismail N., Parker J.A. (2024). Psychobiotics and the Microbiota–Gut–Brain Axis: Where Do We Go from Here?. Microorganisms.

[138] Kim S.-K., Guevarra R.B., Kim Y.T., Kwon J., Kim H., Cho J.H., Kim H.B., Lee J.H. (2019). Role of Probiotics in Human Gut Microbiome-Associated Diseases. J. Microbiol. Biotechnol..

[139] Sánchez B., Delgado S., Blanco-Míguez A., Lourenço A., Gueimonde M., Margolles A. (2017). Probiotics, gut microbiota, and their influence on host health and disease. Mol. Nutr. Food Res..

[140] Gasbarrini G., Bonvicini F., Gramenzi A. (2016). Probiotics History. J. Clin. Gastroenterol..

[141] Dinan T.G., Stanton C., Cryan J.F. (2013). Psychobiotics: A novel class of psychotropic. Biol. Psychiatry.

[142] Sharma R., Gupta D., Mehrotra R., Mago P. (2021). Psychobiotics: The Next-Generation Probiotics for the Brain. Curr. Microbiol..

[143] Ait-Belgnaoui A., Durand H., Cartier C., Chaumaz G., Eutamene H., Ferrier L., Houdeau E., Fioramonti J., Bueno L., Theodorou V. (2012). Prevention of gut leakiness by a probiotic treatment leads to attenuated HPA response to an acute psychological stress in rats. Psychoneuroendocrinology.

[144] Ng Q.X., Peters C., Ho C.Y.X., Lim D.Y., Yeo W.-S. (2018). A meta-analysis of the use of probiotics to alleviate depressive symptoms. J. Affect. Disord..

[145] Tian P., Chen Y., Zhu H., Wang L., Qian X., Zou R., Zhao J., Zhang H., Qian L., Wang Q. (2022). Bifidobacterium breve CCFM1025 attenuates major depression disorder via regulating gut microbiome and tryptophan metabolism: A randomized clinical trial. Brain Behav. Immun..

[146] Schneider E., Doll J.P.K., Schweinfurth N., Kettelhack C., Schaub A.C., Yamanbaeva G., Varghese N., Mählmann L., Brand S., Eckert A. (2023). Effect of short-term, high-dose probiotic supplementation on cognition, related brain functions and BDNF in patients with depression: A secondary analysis of a randomized controlled trial. J. Psychiatry Neurosci..

[147] Yamanbaeva G., Schaub A.C., Schneider E., Schweinfurth N., Kettelhack C., Doll J.P.K., Mählmann L., Brand S., Beglinger C., Borgwardt S. (2023). Effects of a probiotic add-on treatment on fronto-limbic brain structure, function, and perfusion in depression: Secondary neuroimaging findings of a randomized controlled trial. J. Affect. Disord..

[148] Zhang X., Chen S., Zhang M., Ren F., Ren Y., Li Y., Liu N., Zhang Y., Zhang Q., Wang R. (2021). Effects of Fermented Milk Containing Lacticaseibacillus paracasei Strain Shirota on Constipation in Patients with Depression: A Randomized, Double-Blind, Placebo-Controlled Trial. Nutrients.

[149] Casertano M., Dekker M., Valentino V., De Filippis F., Fogliano V., Ercolini D. (2024). Gaba-producing lactobacilli boost cognitive reactivity to negative mood without improving cognitive performance: A human Double-Blind Placebo-Controlled Cross-Over study. Brain Behav. Immun..

[150] Chahwan B., Kwan S., Isik A., van Hemert S., Burke C., Roberts L. (2019). Gut feelings: A randomised, triple-blind, placebo-controlled trial of probiotics for depressive symptoms. J. Affect. Disord..

[151] Rudzki L., Ostrowska L., Pawlak D., Małus A., Pawlak K., Waszkiewicz N., Szulc A. (2019). Probiotic Lactobacillus Plantarum 299v decreases kynurenine concentration and improves cognitive functions in patients with major depression: A double-blind, randomized, placebo controlled study. Psychoneuroendocrinology.

[152] Borkent J., Ioannou M., Neijzen D., Haarman B.C.M., Sommer I.E.C. (2024). Probiotic Formulation for Patients With Bipolar or Schizophrenia Spectrum Disorder: A Double-Blind, Randomized Placebo-Controlled Trial. Schizophr. Bull..

[153] Godzien J., Godzien J., Kalaska B., Rudzki L., Barbas-Bernardos C., Swieton J., Lopez-Gonzalvez A., Ostrowska L., Szulc A., Waszkiewicz N. (2025). Probiotic Lactobacillus plantarum 299v supplementation in patients with major depression in a double-blind, randomized, placebo-controlled trial: A metabolomics study. J. Affect. Disord..

[154] Reininghaus E.Z., Wetzlmair L.C., Fellendorf F.T., Platzer M., Queissner R., Birner A., Pilz R., Hamm C., Maget A., Rieger A. (2020). Probiotic Treatment in Individuals with Euthymic Bipolar Disorder: A Pilot-Study on Clinical Changes and Compliance. Neuropsychobiology.

[155] Antushevich H. (2020). Fecal microbiota transplantation in disease therapy. Clin. Chim. Acta.

[156] Wang J.-W., Kuo C.H., Kuo F.C., Wang Y.K., Hsu W.H., Yu F.J., Hu H.M., Hsu P.I., Wang J.Y., Wu D.C. (2019). Fecal microbiota transplantation: Review and update. J. Formos. Med. Assoc..

[157] Xu H.M., Huang H.L., Zhou Y.L., Zhao H.L., Xu J., Shou D.W., Liu Y.D., Zhou Y.J., Nie Y.Q. (2021). Fecal Microbiota Transplantation: A New Therapeutic Attempt from the Gut to the Brain. Gastroenterol. Res. Pract..

[158] Zhang Q., Bi Y., Zhang B., Jiang Q., Mou C.K., Lei L., Deng Y., Li Y., Yu J., Liu W. (2024). Current landscape of fecal microbiota transplantation in treating depression. Front. Immunol..

[159] Rao J., Qiao Y., Xie R., Lin L., Jiang J., Wang C., Li G. (2021). Fecal microbiota transplantation ameliorates stress-induced depression-like behaviors associated with the inhibition of glial and NLRP3 inflammasome in rat brain. J. Psychiatr. Res..

[160] Cai T., Zheng S.P., Shi X., Yuan L.Z., Hu H., Zhou B., Xiao S.L., Wang F. (2022). Therapeutic effect of fecal microbiota transplantation on chronic unpredictable mild stress-induced depression. Front. Cell. Infect. Microbiol..

[161] Zheng P., Zeng B., Zhou C., Liu M., Fang Z., Xu X., Zeng L., Chen J., Fan S., Du X. (2016). Gut microbiome remodeling induces depressive-like behaviors through a pathway mediated by the host’s metabolism. Mol. Psychiatry.

[162] Settanni C.R., Ianiro G., Bibbò S., Cammarota G., Gasbarrini A. (2021). Gut microbiota alteration and modulation in psychiatric disorders: Current evidence on fecal microbiota transplantation. Prog. Neuro-Psychopharmacol. Biol. Psychiatry.

[163] Guo Q., Lin H., Chen P., Tan S., Wen Z., Lin L., He J., Wen J., Lu S. (2021). Dynamic changes of intestinal flora in patients with irritable bowel syndrome combined with anxiety and depression after oral administration of enterobacteria capsules. Bioengineered.

[164] Wang Y., Zhang S., Borody T.J., Zhang F. (2022). Encyclopedia of fecal microbiota transplantation: A review of effectiveness in the treatment of 85 diseases. Chin. Med. J..

[165] Kang D.W., Adams J.B., Coleman D.M., Pollard E.L., Maldonado J., McDonough-Means S., Caporaso J.G., Krajmalnik-Brown R. (2019). Long-term benefit of Microbiota Transfer Therapy on autism symptoms and gut microbiota. Sci. Rep..

[166] Kwak M., Adams J.B., Coleman D.M., Pollard E.L., Maldonado J., McDonough-Means S., Caporaso J.G., Krajmalnik-Brown R. (2023). Psychobiotics and fecal microbial transplantation for autism and attention-deficit/hyperactivity disorder: Microbiome modulation and therapeutic mechanisms. Front. Cell. Infect. Microbiol..

[167] Green J.E., McGuinness A.J., Berk M., Castle D., Athan E., Hair C., Strandwitz P., Loughman A., Nierenberg A.A., Cryan J.F. (2023). Safety and feasibility of faecal microbiota transplant for major depressive disorder: Study protocol for a pilot randomised controlled trial. Pilot. Feasibility Stud..

[168] Pu Y., Pu Y., Tan Y., Qu Y., Chang L., Wang S., Wei Y., Wang X., Hashimoto K. (2021). A role of the subdiaphragmatic vagus nerve in depression-like phenotypes in mice after fecal microbiota transplantation from Chrna7 knock-out mice with depression-like phenotypes. Brain Behav. Immun..

[169] Angelova I.Y., Kovtun A.S., Averina O.V., Koshenko T.A., Danilenko V.N. (2023). Unveiling the Connection between Microbiota and Depressive Disorder through Machine Learning. Int. J. Mol. Sci..

[170] Jiang Y., Qu Y., Shi L. (2024). The role of gut microbiota and metabolomic pathways in modulating the efficacy of SSRIs for major depressive disorder. Transl. Psychiatry.

[171] Snigdha S., Ha K., Tsai P., Dinan T.G., Bartos J.D., Shahid M. (2022). Probiotics: Potential novel therapeutics for microbiota-gut-brain axis dysfunction across gender and lifespan. Pharmacol. Ther..

[172] Sun Y., Gao S., Ye C., Zhao W. (2023). Gut microbiota dysbiosis in polycystic ovary syndrome: Mechanisms of progression and clinical applications. Front. Cell. Infect. Microbiol..

[173] Li Y., Zhang H., Zheng P., Yang J., Wu J., Huang Y., Hu X., Tan X., Duan J., Chai T. (2022). Perturbed gut microbiota is gender-segregated in unipolar and bipolar depression. J. Affect. Disord..

